# Mid‐childhood developmental and behavioural outcomes in infants with a family history of autism and/or attention deficit hyperactivity disorder

**DOI:** 10.1111/jcpp.70048

**Published:** 2025-09-09

**Authors:** Tony Charman, Tessel Bazelmans, Greg Pasco, Jannath Begum Ali, Mark H. Johnson, Emily J. H. Jones, The BASIS/STAARS Team

**Affiliations:** ^1^ Institute of Psychiatry, Psychology & Neuroscience King's College London London UK; ^2^ Centre for Brain and Cognitive Development Birkbeck, University of London London UK; ^3^ Department of Psychology University of Cambridge Cambridge UK

**Keywords:** Autism, ADHD, family history, infants, mid‐childhood outcomes, behaviour, development

## Abstract

**Background:**

Prospective studies of autism family history infants primarily report recurrence and predictors of autism at 3 years. Less is known about ADHD family history infants and later childhood outcomes. We characterise profiles of mid‐childhood developmental and behavioural outcomes in infants with a family history of autism and/or ADHD to identify potential support needs and patterns of co‐occurrence across domains.

**Methods:**

Two hundred and sixty‐three infants (51% male; *N* = 198 autism/ADHD family history; *N* = 65 no family history) were assessed at 6–12 years. A latent profile analysis (LPA) with indicator variables measuring developmental abilities (IQ, adaptive function) and behavioural traits (autism, ADHD, anxiety) identified dimensional, data‐derived outcome classes.

**Results:**

A seven‐class solution was the most robust and clinically meaningful. Two classes (27% and 23%) had typical development; two classes had high autism, ADHD, and anxiety traits—one with low IQ and adaptive function (10%) and one with average IQ but low adaptive function (13%); one class had elevated autism and ADHD but not anxiety traits (10%); and the final two classes had elevated ADHD (9%) and anxiety (8%) traits in isolation. Sex distribution was balanced across all classes. Children with autism were found in all classes but predominantly in the classes with low IQ/adaptive functioning and high behavioural traits, as well as in the class with elevated autism and ADHD traits. We found only partial continuity between membership of similarly derived 3‐year LPA classes and mid‐childhood LPA classes.

**Conclusions:**

Many autism/ADHD family history infants develop typically. However, by mid‐childhood, in addition to those with autism, others show elevated neurodevelopmental (autism, ADHD) and neuropsychiatric (anxiety) behavioural traits. Lower developmental abilities (IQ and adaptive function) are primarily seen in children with an autism diagnosis. Family history infants should be monitored through childhood, and support provided should challenges emerge.

## Introduction

Autism and ADHD commonly co‐occur within individuals and family members and have shared heritability (Ghirardi et al., 2019; Hollingdale, Woodhouse, Young, Fridman, & Mandy, 2019; Jokiranta‐Olkoniemi et al., 2016, 2019; Miller et al., 2019). Other neurodevelopmental (intellectual disability) and emotional (anxiety, depression) and behavioural (oppositional defiant disorder, conduct disorder) neuropsychiatric conditions also commonly co‐occur with autism (Charman et al., 2011; Lai et al., 2019; Maenner et al., 2023; Simonoff et al., 2008) and ADHD (Elia, Ambrosini, & Berrettini, 2008; Kang et al., 2024; Pliszka, 2000) and within family members with both conditions (Ghirardi et al., 2021; Jokiranta‐Olkoniemi et al., 2016, 2019). Both within‐condition and cross‐condition autism and ADHD associations are also found between family members at the trait level (Ghirardi et al., 2019; Rommelse, Franke, Geurts, Hartman, & Buitelaar, 2010; Van Steijn et al., 2012). Common polygenic variation (autism and ADHD polygenic score (PGS)) is also associated with both early (Askeland et al., 2022) and later (Warrier et al., 2022) emerging neurodevelopmental and neuropsychiatric outcomes. Both within‐condition (e.g. ADHD PGS and ADHD traits) and cross‐condition (e.g. ADHD PGS and autism traits) associations are found, consistent with their shared heritability. In addition to psychiatric conditions, impairments in everyday adaptive functioning are common in both autism and ADHD (Clark, Prior, & Kinsella, 2002; Tillmann et al., 2019). Prospective studies of infants with a family history of neurodevelopmental conditions have the potential to illuminate the earliest emergence of these specific or co‐occurring symptom profiles before they are clinically ascertained, enabling us to inform caregivers and clinicians regarding early signs of atypicality that may require monitoring.

To date, we have a detailed understanding of clinical outcomes in infants with a family history of autism at age 3 (Dawson, Rieder, & Johnson, 2023; Jones, Gliga, Bedford, Charman, & Johnson, 2014; Szatmari et al., 2016). Autism sibling recurrence at 3 years in volunteer research cohorts is ~20% (Ozonoff et al., 2024). In addition to those who have autism, others have lower language, IQ, and adaptive functioning and higher behavioural dysregulation (Charman et al., 2017; Marrus et al., 2018; Messinger et al., 2013; Miller et al., 2019; Ozonoff et al., 2014). The outcomes of infants with a family history of ADHD have been less studied (Johnson, Gliga, Jones, & Charman, 2015; Miller et al., 2023). Miller et al. (2020) used latent profile analysis (LPA) of 3‐year autism and ADHD measures in infants with a family history of ASD and/or ADHD and identified classes with high levels of autism and ADHD scores, respectively, although both classes included children with an autism diagnosis and ‘ADHD concerns’. In a similar cohort, Charman et al. (2023) used LPA on a wider set of measures – also including IQ, adaptive behaviour, and anxiety – and found atypical outcome classes characterised by either low developmental level and high autism, ADHD, and anxiety traits or low adaptive functioning but not elevated behavioural traits. Thus, both a family history of autism and ADHD raise the likelihood of clinically relevant developmental difficulties in toddlerhood. However, we have a limited understanding of whether these early clinical profiles change or persist over later development.

Emerging evidence suggests that following children into mid‐childhood can reveal a range of additional clinical concerns in infant siblings that were not detected at age 3 (Brian et al., 2016; Landa, Reetzke, Holingue, Herman, & Hess, 2022; Ozonoff et al., 2018; Shephard et al., 2017). Bazelmans et al. (2024) reported a sibling autism recurrence rate of 37% in infants with a family history of autism re‐assessed at 9 years. Brian et al. (2016) found a similar mid‐childhood recurrence (34%) but Landa et al. (2022) a lower recurrence rate (20%) at age 6 years. Half of those given an autism diagnosis in mid‐childhood in Bazelmans et al. (2024) were not diagnosed with autism at the earlier 3‐year assessment. Later emerging or later recognised autism is consistent with the recognition in diagnostic manuals that symptoms ‘may not be manifest until social demands exceed limited capacities’ (DSM‐5; American Psychiatric Association, 2022) and is seen in clinical (Davidovitch, Gazit, Patalon, Leitner, & Rotem, 2023; Lord et al., 2006) and population cohorts (Hosozawa et al., 2020; Zhang et al., 2024).

Beyond autism diagnostic recurrence, Miller et al. (2016) found elevated rates of broader autism phenotype and ADHD traits, as well as language and learning difficulties in autism family history infants without autism at 7 years. Shephard et al. (2017) identified elevated anxiety in autism family history infants who themselves did not have an autism diagnosis at 7 years compared with infants with no autism family history. Salomone et al. (2018) reported that autism family history infants both with and without an autism diagnosis had lower IQ than infants with no family history at age 7, but that lower adaptive functioning was primarily found in those with autism. No previous studies have examined the continuity between profiles of preschool and later childhood developmental and behavioural outcomes in autism/ADHD family history infants.

Establishing mid‐childhood profiles of neurodevelopmental and neuropsychiatric outcomes in infants with a family history of autism and/or ADHD – and how they change between preschool and mid‐childhood – will be important both for identifying phenotypic outcomes associated with changes in brain and cognitive development in infant siblings beyond an autism diagnosis and to inform families and clinicians of the support infants may require as they grow up. To address this gap, the current study characterises mid‐childhood developmental and behavioural outcomes in a prospective longitudinal study of autism and/or ADHD family history infants. By design, and in contrast to much of the extant literature (Bazelmans et al., 2024; Brian et al., 2016; Landa et al., 2022), the current analysis does not primarily focus on autism diagnosis as a categorical outcome, although we did characterise autism diagnostic outcomes at both 3 years and mid‐childhood. Instead, we use dimensional measures to characterise developmental and behavioural trait outcomes. We include comparison infants with no family history of autism or ADHD, as well as infants with a family history of autism or ADHD (or both)^1^ to fully utilise the prospective family history design and because these traits are continuously distributed through the population (Martin, Hamshere, Stergiakouli, O'Donovan, & Thapar, 2014; Robinson et al., 2016; Taylor et al., 2019). We conduct an LPA with indicator variables measuring IQ, adaptive function, and autism, ADHD, and anxiety traits to identify data‐derived phenotypic outcome classes and examine their association with family history, sex, and autism diagnosis. We also conduct a similar LPA on 3‐year developmental and behavioural traits to examine the continuity and discontinuity between preschool and mid‐childhood classes.

## Method

### Participants

Four hundred and six infants (213 boys, 193 girls) were enrolled at either 5 or 10 months of age in a prospective family history study if they had a first‐degree relative with a community clinical diagnosis of autism, a community clinical diagnosis of ADHD, or elevated ADHD traits, or both. Parental report of an existing clinical diagnosis of autism and/or ADHD in an older sibling (proband) was the most common route. Some parents reported that they themselves had a diagnosis of either condition, or they or their older child had suspected ADHD, following which screening with a short version of one of the Conners suite of measures was employed to determine eligibility (see Appendix S1 and Table S1 for details). Comparison infants with no autism or ADHD family history and a typically developing older sibling were also recruited. This sample comprised three cohorts: Phase 1 (*n* = 104) reported in Shephard et al. (2017) and new Phase 2 (*n* = 143) and Phase 3 (*n* = 159) cohorts.

Research assessments were undertaken at 5, 10, and 14 months, 2 and 3 years, and mid‐childhood. Age of first visit was 12 months or younger^2^ (*M* (*SD*) = 7.08 (2.21)). The total sample (*N* = 406) comprised 207 infants with a first‐degree relative (family history (FH)) with autism only (FH‐Autism), 30 infants with a first‐degree relative with ADHD only (FH‐ADHD), 67 infants with first‐degree relatives with both autism and ADHD (FH‐Autism + ADHD), and 102 infants with no first‐degree relative with either condition (No‐FH) (Table S1). Of these, 263 infants (64.8%; 133 boys, 130 girls) took part in the mid‐childhood follow‐up at 6–12 years (*M* (*SD*) = 8.65 (1.27)) between July 2014 and December 2023 and had at least one outcome measure used in the LPA and are included in the current analysis: 132 FH‐Autism, 15 FH‐ADHD, 51 FH‐Autism + ADHD, and 65 No‐FH (Table 1). Thirty‐four children did not attend in‐person assessments (parents completed questionnaires only) and an additional seven children attended but an autism diagnostic assessment was not completed, so analyses involving autism diagnostic outcome include 222 children.

**Table 1:** Participant demographic characteristics by family history sampling frame

	FH‐Autism	FH‐Autism + ADHD	FH‐ADHD	No‐FH
	*N* = 132	*N* = 51	*N* = 15	*N* = 65
	*N* (%)	*N* (%)	*N* (%)	*N* (%)
Sex				
Male	64 (48%)	27 (53%)	8 (53%)	34 (52%)
Female	68 (52%)	24 (47%)	7 (47%)	31 (48%)
Age in years Mean (*SD*)	8.83 (1.31)	8.93 (1.35)	8.25 (0.69)	8.13 (1.05)
Child Ethnicity				
White/European/Irish	104 (81%)	43 (86%)	14 (93%)	54 (84%)
Asian/African/African‐Caribbean/Mixed Heritage	25 (19%)	7 (14%)	1 (7%)	10 (16%)
Maternal highest education				
Up to 16/GCSE	13 (10%)	2 (4%)	0 (0%)	2 (3%)
Up to 18/School/College	34 (27%)	17 (35%)	3 (20%)	11 (17%)
Degree level	50 (39%)	16 (33%)	5 (33%)	19 (30%)
Postgraduate/Professional	31 (24%)	13 (27%)	7 (47%)	31 (49%)
Annual household income				
Up to £20,000	11 (9%)	3 (7%)	0 (0%)	4 (7%)
£20,000 to £40,000	32 (26%)	16 (35%)	1 (10%)	9 (15%)
£40,000 to £60,000	38 (31%)	10 (22%)	3 (30%)	13 (22%)
£60,000 to £80,000	19 (15%)	5 (11%)	1 (10%)	9 (15%)
Above £80,000	24 (19%)	12 (26%)	5 (50%)	25 (42%)

Data missing for some demographic variables. FH‐Autism = autism family history, FH‐Autism + ADHD = autism + ADHD family history, FH‐ADHD = ADHD family history, No‐FH = no family history of autism or ADHD.

Ethical approved by NHS RES London REC (14/LO/0170) and King's College London (RESCM‐18/19‐10556). Parents provided written informed consent and children written/verbal assent appropriate to developmental level.

### Measures

#### Family demographic characteristics

##### Ethnicity

Infant sibling ethnicity was characterised as Asian/Black African/Black Caribbean/Mixed versus White/European/Irish.

##### Family sociodemographic information

Annual household income was coded on a 5‐point ordinal scale (<£20,000, £20,000–£40,000, £40,000–£60,000, £60,000–£80,000, >£80,000). Maternal highest education level was coded on a 4‐point ordinal scale (16/GCSE, 18/School/College, Degree level, Postgraduate/Professional).

#### Cognitive ability and adaptive functioning

The *Wechsler Abbreviated Scale of Intelligence – Second Edition* (*WASI‐II*; Wechsler, 2011)^3^ was used to assess full‐scale IQ (FSIQ).^4^ *Vineland‐II* (Phase 1) (Sparrow, Cicchetti, & Balla, 2005) and the *Vineland‐3 Adaptive Behaviour Composite* (ABC) (Phase 2/3) (Sparrow, Cicchetti, & Saulnier, 2016) were used to measure adaptive functioning.

#### Autism diagnostic and screening measures

Observational and parent‐report diagnostic instruments *Autism Diagnostic Observation Schedule‐2* (*ADOS‐2*) (Lord et al., 2012) and *Autism Diagnostic Interview‐Revised* (*ADI‐R*) (Lord, Rutter, & Le Couteur, 1994) and parent‐report *Social Communication Questionnaire* (*SCQ*) (Rutter, Bailey, & Lord, 2003) and *Social Responsiveness Scale‐2* (*SRS‐2*) (Constantino & Gruber, 2012) screening measures were completed.

#### ADHD and anxiety trait measures

Parent‐report *Conners‐3* (Conners, 2008) was used to assess attention deficit hyperactivity disorder (ADHD) symptoms (raw and T‐scores for the DSM‐IV‐TR Inattentive and Hyperactive/Impulsive scales). Parent‐report *Spence Children's Anxiety Scale – Parent* (*SCAS*) (Spence, 1999) was used to assess anxiety symptoms (Total Anxiety raw and T‐scores).

#### Research diagnostic assessments

At both 3 years and mid‐childhood, a best estimate clinical diagnosis of autism spectrum disorder was made based on DSM‐5 criteria.^5^ This was informed by, but not dependent on, scores on the ADOS‐2, ADI‐R, SCQ, and Vineland, researcher observations on the visit, and additional parent‐reported information, by experienced researchers and overseen by a senior clinical psychologist. Diagnosis in mid‐childhood involved review of all previous information, including 2‐ and 3‐year visits, and there was overlap in personnel involved, so decisions were not independent. We previously reported autism sibling recurrence for a subset of the current FH‐Autism infants^6^ (*N* = 159) (Bazelmans et al., 2024). Children with a mid‐childhood autism diagnosis are characterised as those given an autism diagnosis at the 3‐year assessment (*Earlier diagnosed*) and those who were only given an autism diagnosis at the mid‐childhood assessment (*Later diagnosed*).^7^

### Statistical analysis

Retention from first visit to mid‐childhood assessment was examined both univariately using Chi‐squared tests for the binary and ordinal variables and also multivariately using logistic regression (with the Stata logit command) to determine unique predictors of retention accounting for potential collinearity between predictors. Phase, family history, and child (sex, ethnicity) and family (maternal education, family income) factors were tested as predictors of retention. In the univariate tests, we examined both across the four family history groups (FH‐Autism, FH‐Autism + ADHD, FH‐ADHD and No‐FH) and also by looking at a family history of autism (i.e. the FH‐Autism and FH‐Autism + ADHD groups combined) and a family history of ADHD (i.e. the FH‐ADHD and FH‐Autism + ADHD groups combined) separately. In the multivariate logistic regression model, we simultaneously entered all predictors of retention and entered autism family history and ADHD family history treated separately as dummy‐coded (0/1) variables and then their interaction to take account of single and dual family history status.

We conducted LPA using continuous indicator variables to identify homogeneous classes based on the following mid‐childhood indicator variables: WASI FSIQ, Vineland ABC, SRS‐2 total raw score, Conners‐3 Inattention and Hyperactivity/Impulsivity raw scores, and SCAS total anxiety raw score. Variables were modelled, conditional on latent class, using Poisson distributions. LPA was performed using the *gsem* command in Stata 18 (StataCorp., 2023) on the whole sample with at least one of the six outcome measures available (*N* = 263; 240 children had ≥4 measures, 11 ≥ 3, 10 ≥ 2, and 2 children 1 measure only). Models were estimated using maximum likelihood to account for data missing at random. To select the ‘best fitting’ solution, we examined conventional likelihood‐based (Bayesian information criterion (BIC)) and classification‐based (Integrated Classification Likelihood (ICL); entropy) fit statistics (Henson, Reise, & Kim, 2007); the proportion of participants represented in each class; and the extent to which classes captured clinically meaningful subgroups (Nylund, Asparouhov, & Muthén, 2007; Spurk, Hirschi, Wang, Valero, & Kauffeld, 2020). Individuals were assigned to classes based on the maximum aposterior probability of class membership (MAP). The high values of MAP we report made more complex multistep post‐assignment analysis methods unnecessary.

We compared the scores of the outcome classes using ANOVA with post hoc Tukey–Kramer corrections to account for unequal cell sizes and tested sex differences in class assignment using Chi‐square tests. We conducted an LPA analysis on comparable 3‐year measures to examine continuity between 3‐year and mid‐childhood profiles (Appendix S6).

## Results

Retention from recruitment to mid‐childhood is shown in Table S2 for univariate tests and in Table S3 for the multivariate regression model. Univariately, retention was higher in Phases 1 and 2 than in Phase 3 (*p* < .001 and *p* < .05, respectively) and marginally higher in Phase 2 than Phase 3 (*p* = .05). Retention did not differ significantly by family history group (four group test *p* = .079; autism family history and ADHD family history tested separately both *p* > .22), infant sex, maternal education, or family income but was lower in Non‐White families (*p* < .01). In the multivariate model, only Phase significantly independently predicted retention, with the Stata margins pwcompare command indicating that the predicted marginal probabilities of retention were higher in Phase 1 than in Phase 2 (*z* = −2.40, *p* < .05) and in Phase 3 (*z* = −3.39, *p* < .01) taking account of the other predictors.

Table S4 shows mid‐childhood scores on the developmental and behavioural measures by family history group (see Appendix S2).

7‐class and 8‐class solutions had similar entropy values (0.86 and 0.85, respectively) and the best fit statistics (BIC = 11,284.69, ICL = 11,340.44 and BIC = 11,233.91, ICL = 11,287.43, respectively; Table S5). We chose the 7‐class solution as providing the most robust and clinically meaningful distribution of classes, with a minimum class size comprising 8.4% (*N* = 22) of the sample and average MAP values for all classes >0.90. Table S6 shows the correlations between the indicator variables and *R* ^2^ values from regressing each indicator onto the set of classes.

**Figure 1:**
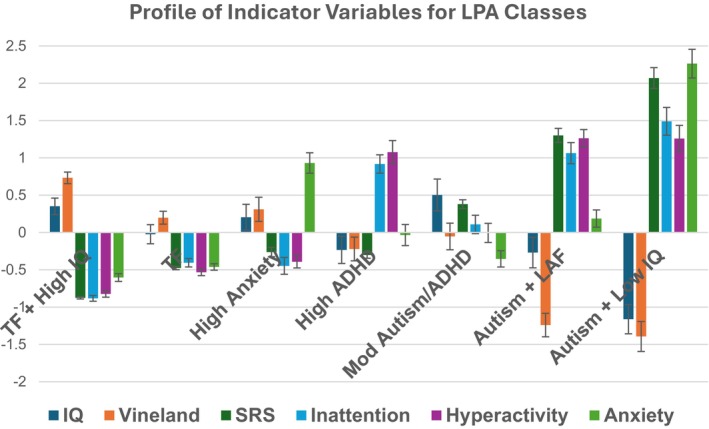
Profile of indicator variables for LPA classes in mid‐childhood. *y*‐axis scale is *z*‐score derived separately for each measure from the current sample so all measures are similarly scaled to provide a profile across the measures. Anxiety, Spence children's anxiety scale‐total; Hyperactivity, Conners hyperactivity/impulsivity; Inattention, Conners inattention; IQ, full‐scale IQ; SRS, social responsiveness scale; Vineland, Vineland adaptive behaviour composite TF + High IQ = typically functioning + High IQ; TF = typically functioning; High Anxiety = high anxiety traits; High ADHD = high ADHD traits; Mod Autism/ADHD = elevated autism + ADHD traits; Autism + LAF = autism + low adaptive Behaviour; Autism + Low IQ = autism + Low IQ

Scores of the 7 classes on the indicator measures used to derive the classes are shown in Table 2 and Figure 1. Based on the pattern across the measures and also the presence versus absence of a mid‐childhood autism diagnosis, we labelled the classes as follows. Class 1 (*N* = 71, 27%) = *Typically Functioning* + *High IQ* (*TF* + *High IQ*) (*N* = 3 (5%) mid‐childhood autism diagnosis^8^); Class 2 (*N* = 61; 23%) = *Typically Functioning* (*TF*) (*N* = 6 (11%) autism); Class 3 (*N* = 22, 8%) = *High Anxiety* (*N* = 2 (10%) autism); Class 4 (*N* = 24, 9%) = *High ADHD* (*N* = 2 (10%) autism); Class 5 (*N* = 25, 10%) = *Moderate Autism/ADHD* (*N* = 10 (50%) autism)^9^; Class 6 (*N* = 34, 13%) = *Autism* + *Low Adaptive Functioning* (*Autism* + *LAF*) (*N* = 23 (82%) autism); and Class 7 (*N* = 26, 10%) = *Autism* + *Low IQ* (*N* = 19 (90%) autism).

**Table 2:** Mid‐childhood scores by LPA classes

	TF + High IQ		TF		High anxiety		High ADHD		Mod autism/ADHD		Autism + LAF		Autism + Low IQ	
	Mean (*SD*)	*N*	Mean (*SD*)	*N*	Mean (*SD*)	*N*	Mean (*SD*)	*N*	Mean (*SD*)	*N*	Mean (*SD*)	*N*	Mean (*SD*)	*N*
Age (months)	101.23 (13.45)	71	101.50 (15.07)	58	112.05 (15.49)	22	101.87 (14.95)	23	109.32 (15.88)	25	102.56 (14.36)	34	106.6 (18.19)	25
WASI FSIQ	115.19 (13.55)_a_	62	109.3 (15.58)_a_	53	112.90 (12.37)_a_	21	106.05 (13.06)_a_	21	117.57 (15.30)_a_	21	105.48 (16.59)_a_	27	91.50 (13.73)_b_	20
Vineland ABC	110.68 (9.45)_a_	63	102.51 (9.39)_c_	51	104.23 (11.61)_a_	22	96.10 (11.15)_c_	21	98.67 (12.50)_c_	21	80.50 (12.26)_b_	26	78.17 (13.04)_b_	18
SRS	41.03 (2.14)_a_	65	47.18 (2.82)_c_	56	50.62 (4.81)_c_	21	50.12 (3.59)_c_	24	60.75 (4.19)_e_	24	74.47 (8.13)_d_	32	87.09 (11.35)_b_	22
Inattention	43.32 (4.49)_a_	66	50.29 (6.72)_c_	59	49.10 (7.82)_a_	21	70.70 (9.53)_b_	23	58.71 (10.30)_d_	24	72.18 (12.16)_b_	33	77.48 (11.92)_b_	21
Hyper‐Imp	44.58 (5.64)_a_	66	48.83 (5.74)_a_	59	52.57 (6.97)_c_	21	75.13 (10.42)_b_	23	58.58 (10.73)_c_	24	77.39 (9.64)_b_	33	76.90 (13.04)_b_	21
Anxiety	46.00 (6.31)_a_	65	48.42 (5.01)_a_	52	63.52 (4.06)_d_	21	53.67 (7.37)_c_	18	49.81 (6.77)_a_	21	56.60 (6.36)_c_	25	68.81 (3.04)_b_	21

Groups marked with different subscript letters (a, b, c) differed significantly with Tukey–Kramer HSD correction applied (*p* < .05). TF + High IQ = typically functioning + High IQ; TF = typically functioning; High Anxiety = high anxiety traits; High ADHD = high ADHD traits; Mod Autism/ADHD = elevated autism + ADHD traits; Autism + LAF = autism + low adaptive Behaviour; Autism + Low IQ = autism + Low IQ. WASI = Wechsler Abbreviated Scale of Intelligence; FSIQ = full‐Scale IQ; ABC = Vineland Adaptive Behaviour Composite; SRS = Social Responsiveness Scale (T‐score); Inattention = Conners Inattention T‐score; Hyper‐Imp = Conners Hyperactivity/Impulsivity T‐score; Anxiety = Spence Children's Anxiety Scale‐Total T‐Score.

As shown in Table 2, ANOVAs and post hocs for class differences are, as expected, in line with class identification (see Appendix S3 for statistical tests). The Autism + Low IQ class had lower FSIQ than the other classes, although the group mean was still in the (low) average range, well below average adaptive functioning, and elevated autism, ADHD, and anxiety trait scores. The Autism + LAF class had average IQ, well below average adaptive functioning, elevated autism and ADHD traits, and somewhat elevated anxiety traits. The Moderate Autism/ADHD class had average IQ and adaptive functioning, and elevated autism and ADHD but not anxiety traits. The High ADHD and High Anxiety classes had average IQ and adaptive functioning, did not have elevated autism traits but showed elevated ADHD and anxiety traits, respectively, in isolation. The TF + High IQ and TF classes both had typical behaviour and average adaptive function. When considered categorically in terms of the proportion of each class falling above or below elevated thresholds for the developmental and behavioural measures (>1*SD*) the pattern was similar (see Table 3).

**Table 3:** Number and percentage of each LPA class in atypical range (±1 *SD*) on mid‐childhood indicator variables

Measure^a^	TF + High IQ	TF	High Anxiety	High ADHD	Mod Autism/ADHD	Autism + LAF	Autism + Low IQ
	*N* = 71	*N* = 61	*N* = 22	*N* = 24	*N* = 25	*N* = 34	*N* = 26
WASI < 85	0 (0%)	2 (4%)	1 (5%)	2 (10%)	0 (0%)	2 (7%)	8 (40%)
Vineland ABC < 85	1 (2%)	1 (2%)	0 (0%)	4 (19%)	3 (14%)	17 (65%)	16 (89%)
SRS T‐score ≥ 60	0 (0%)	0 (0%)	0 (0%)	2 (10%)	12 (50%)	32 (100%)	22 (100%)
Inattention T‐score ≥ 60	1 (2%)	5 (8%)	5 (8%)	3 (14%)	11 (46%)	28 (85%)	20 (95%)
Hyper‐Imp T‐score ≥ 60	2 (3%)	3 (5%)	3 (5%)	2 (10%)	11 (46%)	33 (100%)	18 (86%)
Anxiety T‐score ≥ 60	0 (0%)	0 (0%)	0 (0%)	16 (76%)	2 (23%)	9 (36%)	20 (95%)

TF + High IQ = typically functioning + High IQ; TF = typically functioning; High Anxiety = high anxiety traits; High ADHD = high ADHD traits; Moderate Autism/ADHD = elevated autism + ADHD traits; Autism + LAF = autism + low adaptive behaviour; Autism + Low IQ = autism + Low IQ. WASI = Wechsler Abbreviated Scale of Intelligence; FSIQ = Full‐Scale IQ; ABC = Vineland Adaptive Behaviour Composite; SRS = social Responsiveness Scale (T‐score); Inattention = Conners Inattention T‐Score; Hyper‐Imp = Conners Hyperactivity/Impulsivity T‐score; Anxiety = Spence Children's Anxiety Scale‐Total T‐score.

^a^ Measures available on *N* = 225 WASI, *N* = 222 Vineland, *N* = 244 SRS, *N* = 247 Conners, *N* = 223 SCAS. % are the proportion of those who completed each measure.

Table 4 shows the association between the derived LPA classes and the autism and ADHD family history sampling frame. All children in the Autism + Low IQ class were from the family history groups (all but one from the FH‐Autism or FH‐Autism + ADHD groups), as were most children from the Autism + LAF, Moderate Autism/ADHD, and High Anxiety classes. Three‐quarters (75%) of the No‐FH group were in the TF + High IQ and TF classes. Table 4 also shows the distribution of classes by child sex and presence/absence of an autism diagnosis in mid‐childhood; broken down by earlier versus later diagnosed children. The LPA outcome classes were balanced by sex (*χ* ^2^ (6, *N* = 263) = 1.56, *p* = .96). Children with an autism diagnosis were found in all classes but predominantly in the Autism + Low IQ and Autism + LAF classes and in the Moderate Autism/ADHD class.

**Table 4:** LPA classes by family history group sex and early versus later autism diagnosis^a^

LPA class	No‐FH	FH‐Autism	FH‐ADHD	FH‐Autism + ADHD	Total
	*N* = 65	*N* = 132	*N* = 15	*N* = 51	*N* = 263
	Row %	Row %	Row %	Row %	*N*
	/Column %	/Column %	/Column %	/Column %	
TF + High IQ	32 (45%/49%)	28 (39%/21%)	3 (4%/20%)	8 (11%/16%)	71
TF	17 (28%/26%)	34 (56%/26%)	2 (3%/13%)	8 (13%/16%)	61
High Anxiety	3 (14%/5%)	13 (59%/10%)	0 (0%/0%)	6 (27%/12%)	22
High ADHD	7 (29%/11%)	10 (42%/8%)	2 (8%/13%)	5 (21%/10%)	24
Mod Autism/ADHD	3 (12%/5%)	13 (52%/10%)	6 (24%/40%)	3 (12%/6%)	25
Autism + LAF	3 (9%/5%)	15 (44%/11%)	1 (3%/7%)	15 (44%/29%)	34
Autism + Low IQ	0 (0%/0%)	19 (73%/14%)	1 (4%/7%)	6 (23%/12%)	26

LPA class	Sex	Female	Earlier Autism Diagnosis	Later Autism Diagnosis	Never Autism
	Male				
	*N* = 133	*N* = 130	*N* = 30	*N* = 35	*N* = 157
	*N* (Row %)	*N* (Row %)	*N* (Row %)	*N* (Row %)	*N* (Row %)
TF + High IQ	34 (48%)	37 (52%)	2 (3%)	1 (2%)	58 (95%)
TF	31 (51%)	30 (49%)	1 (2%)	5 (10%)	45 (88%)
High Anxiety	12 (55%)	10 (45%)	1 (5%)	1 (5%)	19 (90%)
High ADHD	11 (46%)	33 (54%)	2 (10%)	0 (0%)	19 (90%)
Mod Autism/ADHD	12 (48%)	13 (52%)	3 (15%)	7 (35%)	10 (50%)
Autism + LAF	20 (59%)	14 (41%)	10 (37%)	13 (48%)	4 (15%)
Autism + Low IQ	13 (50%)	13 (50%)	11 (52%)	8 (38%)	2 (10%)

TF + High IQ = typically functioning + High IQ; TF = typically functioning; High Anxiety = high anxiety traits; High ADHD = high ADHD traits; Mod Autism/ADHD = elevated autism + ADHD traits; Autism + LAF = autism + low adaptive behaviour; Autism + Low IQ = autism + Low IQ. FH‐Autism = autism family history, FH‐Autism + ADHD = autism + ADHD family history, FH‐ADHD = ADHD family history, No‐FH = no‐family history of autism or ADHD.

^a^ Only for the *N* = 222 who underwent both a 3‐year and a mid‐childhood in‐person diagnostic assessment.

**Figure 2:**
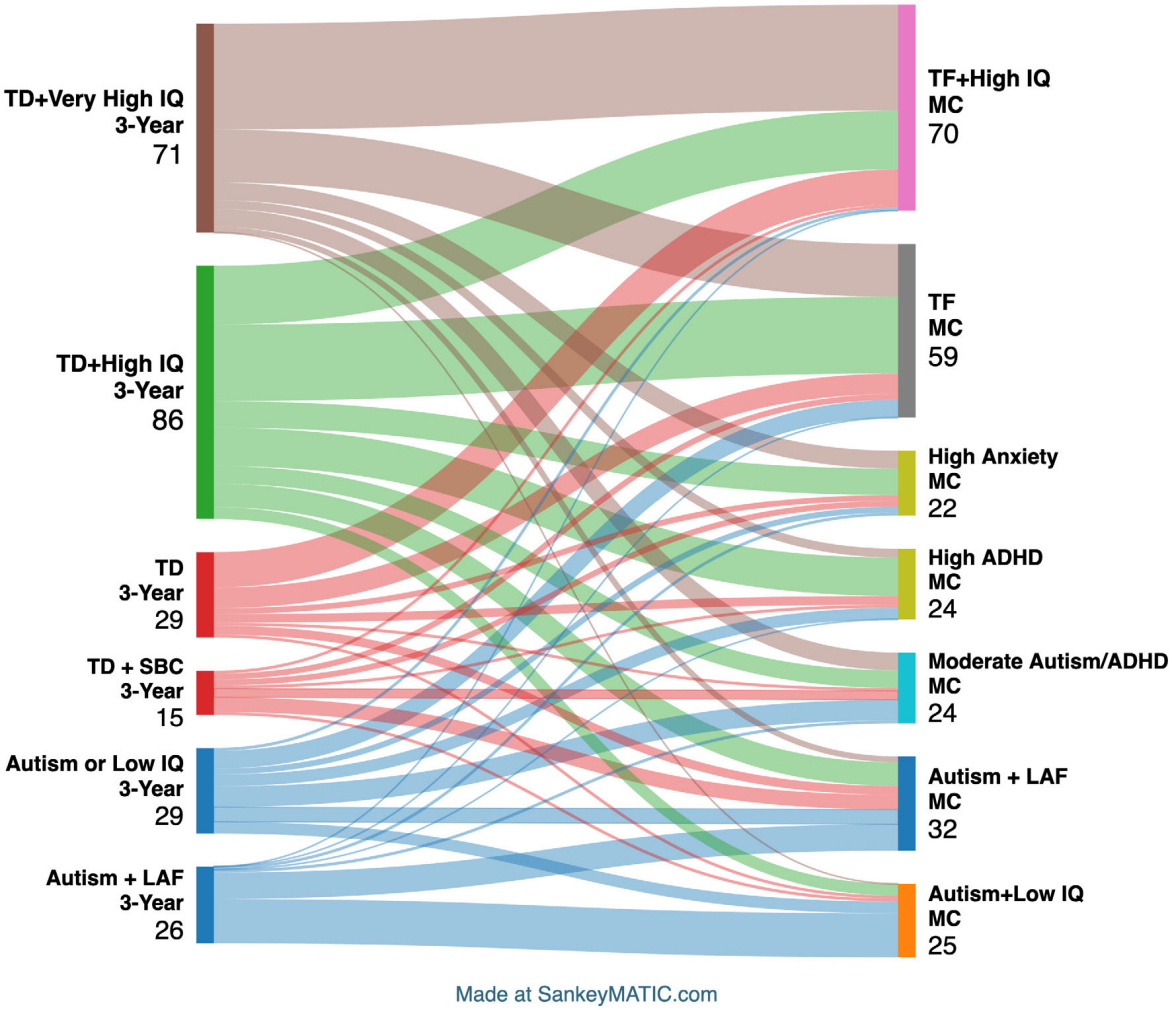
Continuity between 3‐year and mid‐childhood classes. 3‐Year LPA classes: TD + Very High IQ = typically developing + Very High IQ; TD + High IQ = typically developing + High IQ; TD = typically developing; TD + SBC = typical development + some behavioural concerns; Autism or Low IQ; Autism + LAF = autism + low adaptive functioning. Mid‐childhood (MC) LPA classes: TF + High IQ = typically functioning + High IQ; TF = typically functioning; High Anxiety = high anxiety traits; High ADHD = High ADHD traits; Moderate Autism/ADHD = elevated autism + ADHD traits; Autism + LAF = autism + low adaptive behaviour; Autism + Low IQ = autism + Low IQ

Developmental and behavioural scores of the mid‐childhood LPA classes at 3 years and findings from the 3‐year LPA analysis are reported in Appendices S4 and S5 and Tables S7–S10. In brief, the 6‐class solution provided the most robust and clinically meaningful distribution of 3‐year LPA classes. Based on the pattern across the measures and also the presence vs. absence of a 3‐year autism diagnosis, we labelled the classes as follows. Class 1 (*N* = 71, 28%) = *Typically Developing* + *Very High IQ* (*TD* + *Very High IQ*) (2% have autism); Class 2 (*N* = 86, 34%) = *Typical Development* + *High IQ* (*TD* + *High IQ*) (0% have autism); Class 3 (*N* = 29, 11%) = *Typically Developing* (*TD*) (8% have autism); Class 4 (*N* = 15, 6%) = *Typical Development* + *Some Behavioural Concerns* (*TD* + *SBC*) (15% have autism); Class 5 (*N* = 29; 11%) = *Autism or Low IQ* (28% have autism); Class 6 (*N* = 26, 10%) = *Autism* + *Low Adaptive Functioning* (*Autism* + *LAF*) (78% have autism). 3‐year and mid‐childhood LPA class agreement is shown in Figure 2 and Table S11. Continuity between class assignments was strongest for typically developing children and for children with an early (3‐year) autism diagnosis, although a number of children moved from broadly typical to atypical classes and vice versa.

## Discussion

We used a data‐driven approach using dimensional traits measures to examine developmental and behavioural profiles of mid‐childhood outcomes in infants with a family history of autism and/or ADHD. The 7‐class LPA solution we identified, in addition to two classes with high or typical developmental abilities and behaviour, found two classes with low IQ and/or low adaptive function and high autism, ADHD, and anxiety traits and a class with elevated autism and ADHD traits only (with typical IQ and adaptive behaviour and low anxiety traits). Children with a mid‐childhood autism diagnosis were found in all classes but predominantly in these three classes. The final two classes had, respectively, elevated levels of ADHD and anxiety traits in isolation. Sex distribution was balanced across all classes.

The pattern differs in relation to neurodevelopmental (autism, ADHD) and neuropsychiatric (anxiety) *behavioural* outcomes and *developmental* outcomes (IQ, adaptive function). For behavioural traits we identified classes with, respectively, elevated ADHD and anxiety traits in isolation in which very few children had autism and a class with moderately elevated autism and ADHD (but not anxiety) traits, only half of whom were autistic. By contrast, low IQ and/or low adaptive function were only present in the predominantly autistic outcome classes (Autism + Low IQ class: 90% autism; Autism + LAF class: 85% autism) with no other classes and few individual children having below‐average scores. These classes also had high levels not only of autism traits (consistent with their categorical autism diagnosis) but also high levels of ADHD traits, and in the Autism + Low IQ class, high levels of anxiety traits. Overall, our analysis suggests that around half of children with a family history of autism/ADHD might show clinical traits that warrant evaluation and that this extends beyond those who meet diagnostic criteria for autism.

Miller et al. (2016) previously reported elevated levels of autism, ADHD, and anxiety/mood traits in some 7‐year‐old autism family history infants without autism and also lower language abilities. In a sub‐sample of the current cohort, Shephard et al. (2017) found elevated anxiety traits at 7 years in family history infants without autism. The presence of elevated mid‐childhood neurodevelopmental and behavioural traits in autism and ADHD family history infants is consistent with the common co‐occurrence of these in individuals with an autism and ADHD diagnosis and in family members and their shared heritability (Ghirardi et al., 2019, 2021; Hollingdale et al., 2019; Jokiranta‐Olkoniemi et al., 2016, 2019; Lai et al., 2019; Miller, Musser, et al., 2019; Rommelse et al., 2010; Simonoff et al., 2008).

Intellectual disability commonly co‐occurs in autistic individuals (Charman et al., 2011; Maenner et al., 2023) and impairments in adaptive function are common in both autism and ADHD (Clark et al., 2002; Tillmann et al., 2019). In line with this, we found that lower dimensional or trait developmental outcomes aggregated almost exclusively in family history infants with a mid‐childhood autism diagnosis and not in those without. However, our findings differ from previous reports based on 3‐year outcomes that have reported low IQ and adaptive function in some non‐autistic family history infants (Charman et al., 2017, 2023; Marrus et al., 2018; Messinger et al., 2013). Consistent with this, we found only partial continuity between 3‐year and mid‐childhood LPA class membership. Continuity between class assignments was strongest for children with an early (3‐year) autism diagnosis, many of whom at that age also had low IQ and adaptive functioning. Importantly, we note that in our autism/ADHD family history sample very few children had low IQ, with only 15 (7%) scoring <85 (Table 3) and only 5 (2%) scoring <70. This is reflected in the below‐average mean score of the class labelled Autism + Low IQ, whose mean IQ was 91.50 (*SD* = 13.73). In line with this, only 3 children from 223 who completed ADOS‐2 assessments were not able to do Module 3's that require fluent verbal speech.^10^ However, both the Autism + Low IQ class and the Autism + LAF class had mean adaptive scores well below average (*M* = 78.17 (*SD* = 13.04) and *M* = 80.50 (*SD* = 12.26), respectively – see Table 2), demonstrating the well‐established pattern that many individuals with autism have everyday adaptive functional skills lower than their scores on cognitive tests (Tillmann et al., 2019). A similar pattern with relatively lower IQ/DQ and language scores at 3 years but low average range IQ scores in mid‐childhood has also recently been reported in another study of autism family history infants (Gangi et al., 2025).

The most notable discontinuity was that a proportion of children identified in the TD + Very High IQ and TD + High IQ classes at 3 years went on by mid‐childhood to be identified in the Autism + LAF, Moderate Autism/ADHD, and High ADHD and High Anxiety classes. This pattern is consistent with our finding for categorical autism diagnostic outcome where a significant proportion of children who met criteria for autism at the mid‐childhood assessment were not given an autism diagnosis at 3 years (Bazelmans et al., 2024). The presence of neurodevelopmental and neuropsychiatric conditions changes as children develop. This is true both in the general population (Finsaas, Bufferd, Dougherty, Carlson, & Klein, 2018) and in autistic children (Hollocks et al., 2023; Wiggins et al., 2024). Our study confirms that this is also true for children with a family history of autism and ADHD, necessitating both characterising wider outcomes beyond autism and ADHD and continuing to clinically monitor children in this group beyond the preschool period.

All classes were balanced for sex. Previous autism family history studies have reported a more balanced 3‐year autism diagnosis sex ratio (~2:1) than population prevalence studies (Maenner et al., 2023; Ozonoff et al., 2024; Zwaigenbaum et al., 2012) and this was also true at mid‐childhood in our cohort (Bazelmans et al., 2024). One possibility is that our dimensional data‐driven approach to classifying outcomes identifies girls expressing similar levels of autism traits to boys who, despite this, would not meet the threshold for an autism diagnosis. This has previously been identified in both autism family history and in general population studies that have used data‐driven approaches to examine sex differences in autism traits (Burrows et al., 2022; Russell, Steer, & Golding, 2011). The classes with elevated ADHD and anxiety traits only were also balanced for sex. This contrasts with population studies that show higher rates of ADHD diagnosis in boys than girls prior to adolescence (13 years) but higher rates of anxiety disorders in girls only from 13 to 18 years (Dalsgaard et al., 2020) which may not have been seen at 6–12 years in this group.

### Strengths and limitations

The study has a number of strengths. We followed a moderate‐to‐large sample of autism and ADHD family history infants recruited in the first year of life to mid‐childhood. Few studies to date have followed autism/ADHD family history infants beyond 3 years, and both neurodevelopmental and neuropsychiatric outcomes can be more reliably measured in mid‐childhood (Finsaas et al., 2018). Extending beyond previous studies primarily reporting autism recurrence, we characterise broader developmental and behavioural outcomes using a parsimonious person‐centred data‐driven modelling approach.

However, there are a number of limitations. Although we include both autism and ADHD family history infants (as well as no family history controls) the autism family history group (both those with and without an ADHD family history) comprises the largest group. We conducted autism diagnostic assessments but did not do so for ADHD and anxiety, reporting trait measures only. There is a reliance on parental report measures across all constructs except IQ, resulting in potential shared methods variance limitations and other potential biases, including the extent to which questionnaire measures can reliably distinguish between different psychopathology traits (Hus, Bishop, Gotham, Huerta, & Lord, 2013). Finally, our volunteer research sample had above‐average sociodemographic characteristics (family income and maternal education) and findings may not generalise to the wider population. We did test factors related to retention/attrition from first visit to mid‐childhood, and in a parsimonious multivariate model, only Phase (from our three sequential cohorts) was systematically associated with retention, while family income, maternal education, child ethnicity, and family history group were not. We do not have an obvious explanation for this cohort effect. It is important to recognise that research participants in family history studies may have particular motivations to volunteer and other hard‐to‐measure parent, family, and child factors that may impact representativeness and outcomes in addition to the above‐average sociodemographic characteristics we have identified.

## Conclusions

Infant family history studies may provide unique insights into the structure of psychopathology. Their prospective nature allows us to test infant neurocognitive and behavioural predictors of later outcomes to reveal underlying mechanisms of neurodivergent versus typical development (Dawson et al., 2023; Johnson et al., 2015; Jones et al., 2014; Szatmari et al., 2016). Recruitment is based on family history and not clinical presentation and may reveal the natural history both of the timing of emergence of atypicality and of the covariation between neurodevelopmental and neuropsychiatric traits in autism and ADHD family members.

Many infants with a family history of autism and/or ADHD develop typically. However, by mid‐childhood, in addition to those who have autism (Bazelmans et al., 2024), around one‐third of infants with a family history of autism/ADHD have elevated neurodevelopmental (autism, ADHD) and neuropsychiatric (anxiety) traits. By contrast, lower developmental outcomes (IQ and adaptive function) primarily co‐occur in history infants with a family history of an autism diagnosis. Parents and clinicians need to be aware of this range of outcomes – beyond categorical autism diagnosis – as family history infants grow up, and support provided should challenges emerge. Measuring wider neurodevelopmental and behavioural outcomes also provides an opportunity for prospective autism and ADHD family history studies to examine the transdiagnostic specificity (Astle, Holmes, Kievit, & Gathercole, 2022; Michelini et al., 2024; Thapar, Cooper, & Rutter, 2017) of early neurobiological, neurocognitive and behavioural markers to later outcomes (Gui et al., 2020; Johnson et al., 2015; Shephard et al., 2019) which may identify novel translational insights and opportunities.

## Ethical considerations

Parents provided written informed consent and children written/verbal assent appropriate to developmental level. Ethical approval from NHS Health Research Authority, National Research Ethics Service (NRES Committee London Central, 14/LO/0170, 13/05/2014) and Psychiatry, Nursing and Midwifery Research Ethics Subcommittee, King's College London (RESCM‐18/19‐10556, 28/03/2019).

**Key points**

**What's known?**

- Prospective studies of autism family history infants primarily report on autism recurrence and predictors of autism at 3 years but less is known about later childhood outcomes and ADHD family history infants.

**What's new?**

- By mid‐childhood, in addition to those with an autism diagnosis, around one‐third of infants with a family history of autism and/or ADHD have elevated autism, ADHD, and anxiety traits.
- However, low IQ and adaptive functioning primarily co‐occur in those with an autism diagnosis.
- There was only partial continuity between 3‐year and mid‐childhood profiles, with some children being identified in typical classes at 3 years who went on to show elevated autism, ADHD, and anxiety traits in mid‐childhood.

**What's relevant?**

- Family history infants should be monitored through childhood, and support provided when challenges emerge.

## Supporting information


**Appendix S1.** Sample recruitment and characterization.
**Table S1.** Categorisation of family history group.
**Table S2.** Sample descriptives of retained vs. non‐retained from recruitment to mid‐childhood.
**Table S3.** Summary of multivariate logistic regression analysis for retention from recruitment to mid‐childhood.
**Table S4.** Mid‐childhood scores by family history group.
**Appendix S2.** Summary of mid‐childhood scores on the developmental and behavioural measures by family history group.
**Table S5.** Summary of the latent profile analysis (LPA) models.
**Table S6.** Correlations between indicator variables and regression of indictor variables on LPA 7‐class solution.
**Appendix S3.** Statistical analysis of mid‐childhood LPA classes on indicator variables.
**Table S7.** Three year scores by mid‐childhood LPA classes.
**Appendix S4.** Developmental and behavioural characterisation of the mid‐childhood LPA classes at 3‐years.
**Appendix S5.** Statistical analysis of 3‐year scores by mid‐childhood LPA classes.
**Appendix S6.** 3‐year LPA analysis.
**Table S8.** 3‐years scores by 3‐year LPA classes.
**Table S9.** 3‐year LPA class by family history group.
**Table S10.** 3‐year LPA classes by sex and mid‐childhood autism diagnosis (early vs. later).
**Table S11.** 3‐year LPA class by mid‐childhood LPA class.
**Appendix S7.** Mid‐childhood LPA repeated for autism and/or ADHD family history infants only.
**Table S12.** Mid‐childhood scores by LPA classes for family history infants only.
**Table S13.** Mid‐childhood classes for family history infants only LPA by family history group.
**Table S14.** Mid‐childhood classes for family history infants only LPA by autism diagnosis and by sex.

## Data Availability

Data available following review as indicated here: https://www.basisnetwork.org/collaboration‐and‐project‐affiliation/.

## Endnotes

1 We report results a similar LPA in the autism and/or ADHD family history infants only (excluding the No‐FH infants) in the Supplementary Materials.

2 Six children's first research visit was after 12 months (13–15 months).

3 4 subtest version (Block design, Vocabulary, Matrix Reasoning, Similarities) in Phase 1 and 2‐subtest (Vocabulary, Matrix Reasoning) version in Phases 2 and 3.

4 On the *N* = 229 children seen for in‐person assessments WASIs were completed on *N* = 225. Only two children were unable to access the WASI due to limited language ability.

5 In the Phase 1 cohort 3‐year diagnostic was assigned using ICD‐10 but on subsequent review all cases who met criteria for pervasive developmental disorder met DSM‐5 criteria for autism spectrum disorder.

6 Including those who also had an ADHD Family History (FH‐Autism + ADHD).

7 Excluding the *N* = 3 who ‘lost diagnosis’ between the 3‐year and mid‐childhood diagnostic assessment (see Bazelmans et al., 2024; Shephard et al., 2017).

8
*N* (%) with a mid‐childhood autism diagnosis relates to the subset (*N* = 222) on whom full autism diagnostic assessments were completed (see Method).

9 The LPA including autism and/or ADHD family history infants only produced a similar 6‐class solution with two typically developing/functioning classes, two predominantly autism classes (one with low IQ and low adaptive functioning, one low adaptive functioning), and two classes with, respectively, predominantly isolated ADHD and anxiety traits in isolation. Children with an autism diagnosis were found in all classes (see Appendix S7 and Tables S12–S14).

10 One child completed a Module 1 (preverbal/single words) and two children a Module 2 (short phrase speech).
