# Supplementary material for: Mid‐childhood developmental and behavioural outcomes in infants with a family history of autism and/or attention deficit hyperactivity disorder
Source: J Child Psychol Psychiatry. 2025 Sep 9;67(2):282–95. doi: 10.1111/jcpp.70048 (PMC12812788; doi:10.1111/jcpp.70048)
Supplement: Supplementary file 1 — Appendix S1. Sample recruitment and characterization. Table S1. Categorisation of family history group. Table S2. Sample descriptives of retained vs. non‐retained from recruitment to mid‐childhood. Table S3. Summary of multivariate logistic regression analysis for retention from recruitment to mid‐childhood. Table S4. Mid‐childhood scores by family history group. Appendix S2. Summary of mid‐childhood scores on the developmental and behavioural measures by family history group. Table S5. Summary of the latent profile analysis (LPA) models. Table S6. Correlations between indicator variables and regression of indictor variables on LPA 7‐class solution. Appendix S3. Statistical analysis of mid‐childhood LPA classes on indicator variables. Table S7. Three year scores by mid‐childhood LPA classes. Appendix S4. Developmental and behavioural characterisation of the mid‐childhood LPA classes at 3‐years. Appendix S5. Statistical analysis of 3‐year scores by mid‐childhood LPA classes. Appendix S6. 3‐year LPA analysis. Table S8. 3‐years scores by 3‐year LPA classes. Table S9. 3‐year LPA class by family history group. Table S10. 3‐year LPA classes by sex and mid‐childhood autism diagnosis (early vs. later). Table S11. 3‐year LPA class by mid‐childhood LPA class. Appendix S7. Mid‐childhood LPA repeated for autism and/or ADHD family history infants only. Table S12. Mid‐childhood scores by LPA classes for family history infants only. Table S13. Mid‐childhood classes for family history infants only LPA by family history group. Table S14. Mid‐childhood classes for family history infants only LPA by autism diagnosis and by sex. [file JCPP-67-282-s001.docx]

**01-08-2025**

SUPPLEMENTARY MATERIALS

Mid-Childhood Developmental and Behavioural Outcomes in Infants with a Family History of Autism and / or Attention Deficit Hyperactivity Disorder (ADHD)

**Appendix S1**

**Sample Recruitment and Characterization**

Four hundred and six infant siblings (213 boys, 193 girls) were recruited in a prospective family history study. Family history infants at either 5 or 10 months of age were enrolled if they had a first-degree relative with a community clinical diagnosis of autism, a first-degree relative with a community clinical diagnosis of ADHD or elevated ADHD traits, or both.

Participants were recruited for a longitudinal study running from 2013 to 2024 from a volunteer database, community flyers, internet adverts and clinical networks. Parental report of an existing community clinical diagnosis of autism and / or ADHD in an older sibling (proband) was the most common route (see Table S1 below). Some parents reported that they themselves had a community clinical diagnosis of either condition or they or their older child had suspected ADHD following which screening with a short version of one the Conners suite of measures was employed to determine eligibility (see below). Information about diagnostic status was ascertained through a number of methods. Before families enrolled in the study, a telephone screening was conducted to determine the presence of autism and ADHD in family members. During their infant’s visit to the lab, the parent/caregiver also completed a medical and psychiatric history interview. We asked for medical updates at each subsequent study visit including new autism and ADHD diagnoses in family members. We also requested diagnostic letters and asked parents to complete the DAWBA (Goodman et al., 2000), the Conners (Conners, 2008) and the Social Communication Questionnaire (Rutter et al., 2003) on the older sibling (proband) with an autism or ADHD diagnosis and where possible other siblings. This information is used to characterise our sample rather than for exclusionary purposes since in the UK NHS clinical diagnoses follow multidisciplinary assessments involving collation of information from parents, teachers and from in-person assessment that is beyond the scope of this study and more valid than questionnaire measures.

Up to 30% of children with autism meet criteria for ADHD when directly assessed (Simonoff et al., 2008). In clinical practice, the prevalence of dual diagnosis is in practice lower (Russell et al., 2014). Given the nature of the co-occurrence between autism and ADHD and our longitudinal study, sometimes family members would have a suspected diagnosis of ADHD at study entry that would be confirmed later in the study; on other occasions, a family would enrol on the basis of an autism diagnosis in an older sibling but by the end of the study, they would report that the same sibling was now undergoing assessment for suspected additional ADHD. At initial contact with participants, parents were asked if there were any diagnoses of ADHD in the immediate family or if they had any concerns about ADHD. If parents reported concerns about suspected ADHD at study entry, they were screened using a shortened version of the Conners to determine eligibility for the study. For siblings aged less than 6 years a shortened version of the Conners Early Childhood (Conners, 2009) and for siblings 6 years or older a shortened version of the Conners 3 (Conners, 2008) were used. Thresholds for inclusion were the presence of 6 ADHD traits on either the hyperactivity/impulsivity or inattention scale, and a positive score on the impairment scale. For parents a shortened version of the Conners Adults ADHD Rating Scale (CAARS; (Conners et al., 1999)) was used. Thresholds for inclusion were the presence of 5 ADHD traits on either the hyperactivity/impulsivity or inattention scale as per DSM-5 guidelines. In terms of the use impairment scores, we used a reduced version of the Conners EC and Conners 3 for individuals under 18 and the CAARS for individuals aged 18+ years. The Conners EC and Conners 3 included questions regarding impairment, as such we also included these questions in our screening forms. In comparison, the CAARS (adult questionnaire) did not include questions regarding impairment. In order to maintain consistency of measure, we did not adapt the CAARS to add impairment questions. As shown below in Table S1, this was the case only for a small minority of participants in the Family History ADHD group and for many children initially suspected ADHD was subsequently confirmed as a diagnosis by parental report on review at the mid-childhood follow-up. This resulted in most diagnoses and hence allocation to Family History group being based on a parent report of a confirmed community clinical diagnosis of an older sibling or parent.

Parent-reported family medical histories were examined for significant conditions in the proband or extended family members (e.g., Fragile X syndrome, tuberous sclerosis) with no such conditions reported. 384 infants were born full-term (37-42 weeks), 14 infants were preterm (32-36 weeks) and term/gestation data was missing for 8 infants. Infants were (*M (SD)* = 7.08 (2.21)) months old (range 3 to 15 months) at their first infant research assessment with all but 6 children’s first research visit being at or before 12 months.

**Table S1** *Categorisation of Family History Group*

|  | FH-Autism  *N* = 207 | FH-ADHD  *N* = 30 | FH-Autism + ADHD  *N* = 67 | No FH  *N* = 102 |
| --- | --- | --- | --- | --- |
| Parent reported diagnosis in older sibling | 196^a^ | 12^b^ | 50 |  |
| Parent reported diagnosis in parent | 1 | 17 | 2 |  |
| Parent reported diagnosis in both older sibling and parent | 10 | 1 | 15 |  |
| Screened parent (for ADHD traits) |  | 0 | 3^c^ |  |
| Screened older sibling (for ADHD traits) |  | 0 | 2^d^ |  |

FH-Autism = Autism Family History, FH-Autism + ADHD = Autism + ADHD Family History, FH-ADHD = ADHD Family History, No FH = No Family History of Autism or ADHD

a = 5 half-siblings; b = 2 half-siblings; c = Sibling diagnosed with autism, parent also screened positive for ADHD traits; d = Older sibling with autism, sibling also screened positive for ADHD traits.

**Table S2** *Sample Descriptives of Retained vs. Non-Retained from Recruitment to Mid-childhood*

Retained (*N* = 263) Non-Retained (*N* = 143)

Recruitment Count (Row %) Count (Row %) *X*^2^ (p-value)

Phase

1 81 (78%) 23 (22%) *X*^2^ (2, *N* = 406) = 15.06, *p* = .001

2 95 (66%) 48 (34%)

3 87 (55%) 72 (45%)

FH Group

FH-Autism 132 (64%) 75 (36%) *X*^2^ (3, *N* = 406) = 6.79, *p* = .079

FH-Autism + ADHD 51 (76%) 16 (24%)

FH-ADHD 15 (50%) 15 (50%)

No FH 65 (64%) 37 (36%)

Autism FH^a^ 183 (67%) 91 (33%) *X*^2^ (1, *N* = 406) = 1.49, *p* = .222

No Autism FH 80 (61%) 52 (39%)

ADHD FH^b^ 66 (68%) 31 (32%) *X*^2^ (1, *N* = 406) = 0.59, *p* = .441

No ADHD FH 197 (64%) 112 (36%)

Child Sex

Male 133 (62%) 80 (38%) *X*^2^ (1, *N* = 406) = 1.07, *p* = .300

Female 130 (67%) 63 (33)%

Child Ethnicity

Asian/African/Black/African

Caribbean/Mixed 43 (52%) 39 (48%) *X*^2^ (1, *N* = 390) = 8.72, *p* = .003

White/European/Irish 215 (70%) 93 (30%)

Annual Household Income

Up to £20,000 18 (64%) 10 (36%) *X*^2^ (4, *N* = 342) = 2.82, *p* = .588

£20,000 to £40,000 58 (65%) 31 (35%)

£40,000 to £60,000 64 (72%) 25 (28%)

£60,000 to £80,000 34 (77%) 10 (23%)

Above £80,000 66 (72%) 26 (28%)

Maternal Highest Education

Up to 16/GCSE 17 (71%) 7 (29%) *X*^2^ (3, *N* = 383) = 2.60, *p* = .457

Up to 18/School/College 65 (60%) 43 (40%)

Degree level 90 (68%) 42 (32%)

Postgraduate/Professional 82 (69%) 37 (31%)

a Family history of autism (i.e. the FH-Autism and FH-Autism + ADHD groups combined)

b Family history of ADHD (i.e. the FH-ADHD and FH-Autism + ADHD groups combined)

**Table S3** *Summary of Multivariate logistic regression analysis for retention from recruitment to mid-childhood*

| \|  \| *Coefficient* \| *SE* \| *t* \| *p* \| *[95% conf. interval]* \| \| \| --- \| --- \| --- \| --- \| --- \| --- \| --- \| \|  \|  \|  \|  \|  \|  \|  \| \| Phase (1) \|  \|  \|  \|  \|  \|  \| \| **2** \| **.46** \| **.16** \| **-2.28** \| **0.023** \| **.23** \| **.90** \| \| **3** \| **.31** \| **.11** \| **-3.18** \| **0.001** \| **.15** \| **.64** \| \|  \|  \|  \|  \|  \|  \|  \| \| Autism Family History \| 1.49 \| .48 \| 1.25 \| 0.210 \| .80 \| 2.79 \| \|  \|  \|  \|  \|  \|  \|  \| \| ADHD Family History \| 2.88 \| 2.14 \| 1.43 \| 0.154 \| .67 \| 12.32 \| \|  \|  \|  \|  \|  \|  \|  \| \| Autism-FH * ADHD-FH \| .58 \| .48 \| -0.66 \| 0.511 \| .12 \| 2.90 \| \|  \|  \|  \|  \|  \|  \|  \| \| Child Sex \| 1.13 \| .28 \| 0.47 \| 0.635 \| .69 \| 1.84 \| \|  \|  \|  \|  \|  \|  \|  \| \| Ethnicity \| 1.60 \| .48 \| 1.58 \| 0.115 \| .89 \| 2.88 \| \|  \|  \|  \|  \|  \|  \|  \| \| Maternal Education \| 1.08 \| .16 \| 0.51 \| 0.611 \| .81 \| 1.43 \| \|  \|  \|  \|  \|  \|  \|  \| \| Family Income \| 1.15 \| .12 \| 1.31 \| 0.190 \| .93 \| 1.40 \| \|  \|  \|  \|  \|  \|  \|  \| \| Constant \| 1.09 \| .66 \| 0.15 \| 0.882 \| .33 \| 3.58 \| \|  \| *N* = 332, *F* = 19.56, *p* = .021 \| \| \| \| \| \| |
| --- | --- | --- | --- | --- | --- | --- | --- | --- | --- | --- | --- | --- | --- | --- | --- | --- | --- | --- | --- | --- | --- | --- | --- | --- | --- | --- | --- | --- | --- | --- | --- | --- | --- | --- | --- | --- | --- | --- | --- | --- | --- | --- | --- | --- | --- | --- | --- | --- | --- | --- | --- | --- | --- | --- | --- | --- | --- | --- | --- | --- | --- | --- | --- | --- | --- | --- | --- | --- | --- | --- | --- | --- | --- | --- | --- | --- | --- | --- | --- | --- | --- | --- | --- | --- | --- | --- | --- | --- | --- | --- | --- | --- | --- | --- | --- | --- | --- | --- | --- | --- | --- | --- | --- | --- | --- | --- | --- | --- | --- | --- | --- | --- | --- | --- | --- | --- | --- | --- | --- | --- | --- | --- | --- | --- | --- | --- | --- | --- | --- | --- | --- | --- | --- | --- | --- | --- | --- | --- | --- | --- | --- | --- | --- | --- | --- | --- | --- | --- | --- | --- | --- | --- | --- | --- |

| **Table S4**  *Mid-Childhood Scores by Family History Group* | | | | | | | | | | | | |
| --- | --- | --- | --- | --- | --- | --- | --- | --- | --- | --- | --- | --- |
|  | Family History Group | | | | | | | | | | | |
|  | No FH | | | FH-ASD | | | FH-ADHD | | | FH-ASD + ADHD | | |
|  | *Mean* | *SD* | *N* | *Mean* | *SD* | *N* | *Mean* | *SD* | *N* | *Mean* | *SD* | *N* |
| Age (months) | 97.58_a_ | (12.62) | 64 | 106.00_b_ | (15.76) | 130 | 99.00 | (8.22) | 14 | 107.12_b_ | (16.15) | 50 |
| WASI FSIQ | 114.57_a_ | (12.85) | 60 | 107.21_b_ | (15.94) | 117 | 122.17 | (13.45) | 6 | 107.90 | (16.81) | 42 |
| Vineland ABC | 107.88_a_ | (10.73) | 59 | 96.59_b_ | (14.41) | 116 | 106.00 | (20.36) | 6 | 94.61_b_ | (17.84) | 41 |
| SRS T-score | 45.78_a_ | (8.45) | 63 | 57.05_b_ | (16.65) | 118 | 54.67 | (11.99) | 15 | 60.33_b_ | (16.77) | 48 |
| Conners Inattention T-score | 50.37_a_ | (10.15) | 65 | 57.82_b_ | (15.26) | 121 | 57.13 | (11.98) | 15 | 60.30_b_ | (17.82) | 46 |
| Conners Hyper-Imp T-score | 51.57_a_ | (12.50) | 65 | 58.42_b_ | (15.70) | 121 | 60.87 | (13.05) | 15 | 62.96_b_ | (18.02) | 46 |
| SCAS Anxiety T-score | 47.15_a_ | (7.26) | 61 | 54.62_b_ | (9.38) | 109 | 49.92 | (9.15) | 13 | 55.88_b_ | (8.85) | 40 |

**Note:** Groups marked with different subscript letters (a, b) differed significantly with Tukey-Kramer correction applied (p < .05).

WASI = Wechsler Abbreviated Scale of Intelligence; FSIQ = Full Scale IQ; ABC = Vineland Adaptive Behavior Composite, SRS = Social Responsiveness Scale, SCAS = Spence Children’s Anxiety Scale

**Appendix S2**

**Summary of mid-childhood scores on the developmental and behavioural measures by family history group**

As shown in Table S3, Mid-childhood IQ was highest in the No FH group and Vineland adaptive behaviour scores were lower in the FH-ASD and FH-ASD + ADHD groups compared to the No FH group. Scores on the autism, ADHD and anxiety trait measures were higher in all three FH groups compared to the No FH group, although these differences were not statistically significant for the FH-ADHD group, likely due to small sample size.

We analysed group differences by categorical ANOVA with FH-Autism (0/1) and FH-ADHD (0/1) and their interaction (FH-Autism * FH-ADHD) as independent variables and mid-childhood developmental and behavioural score as dependent variable. For FSIQ the overall ANOVA was significant (﻿*F*(3, 221) = 4.58, *p* < .01) and the effect of FH-Autism was significant (*F*(1, 221) = 9.27, *p* < .01) but the effect of FH-ADHD and the FH-Autism * FH-ADHD were not (both *p* > .24). For Vineland ABC the overall ANOVA was significant (﻿*F*(3, 218) = 10.20, *p* < .001) and the effect of FH-Autism was significant (*F*(1, 218) = 11.42, *p* < .001) but FH-ADHD and FH-Autism * FH-ADHD were not (both *p* > .56). For SRS-2 the overall ANOVA was significant (﻿*F*(3, 240) = 11.04, *p* < .001) and FH-Autism (*F*(1, 240) = 11.82, *p* < .001) and FH-ADHD (*F*(1, 240) = 6.10, *p* < .05) were significant but FH-Autism * FH-ADHD was not (*p* = .26). For Conners-Inattention the overall ANOVA was significant (﻿*F*(3, 243) = 5.28, *p* < .01) and FH-Autism (*F*(1, 243) = 4.81, *p* < .05) was significant and FH-ADHD (*F*(1, 243) = 3.65, *p* = .06) a non-significant trend but FH-Autism * FH-ADHD was not (*p* = .38). For Conners-Hyperactivity/Impulsivity the overall ANOVA was significant (﻿*F*(3, 243) = 5.62, *p* < .01) and FH-Autism (*F*(1, 243) = 3.07, *p* = .08) was a nonsignificant trend and FH-ADHD (*F*(1, 243) = 7.34, *p* < .01) was significant but FH-Autism * FH-ADHD was not (*p* = .35). For SCAS Anxiety the overall ANOVA was significant (﻿*F*(3, 219) = 12.14, *p* < .001) and the effect of FH-Autism was significant (*F*(1, 219) = 18.53, *p* < .001) but FH-ADHD and FH-Autism * FH-ADHD were not (both *p* > .19).

**Table S5** *Summary of the Latent Profile Analysis (LPA) Models*

Classes Entropy BIC^a^ ICL^b^ Relative prevalence MAP^c^

1 2 3 4 5 6 7 8 1 2 3 4 5 6 7 8

5 .89 11647.22 11685.41 .27 .12 .29 .12 .20 - - - .96 .95 .94 .96 .97 - - -

6 .87 11414.72 11462.20 .26 .11 .25 .12 .11 .15 - - .93 .95 .92 .95 .96 .98 - -

**7 .86 11284.69 11340.44 .26 .10 .24 .09 .12 .08 .10 - .93 .94 .93 .96 .95 .90 .97 -**

8 .85 11233.91 11287.43 .26 .02 .09 .24 .11 .09 .08 .10 .93 .91 .96 .93 .93 .92 .90 .97

a BIC = Bayesian Information Criterion, b ICL = Integrated Classification Likelihood, c Maximum aposterior probability of class membership

**Bold solution was chosen as providing the most robust and clinically meaningful distribution of classes**

**Table S6** *Correlations between Indicator Variables and Regression of Indictor Variables on LPA 7-Class Solution*

Vineland SRS Inattention Hyper-Imp Anxiety R-squared

WASI 0.45^***^ -0.29^***^ -0.29^***^ -0.21^**^ -0.24^***^ 0.17^***^

Vineland ABC -0.67^***^ -0.61^***^ -0.49^***^ -0.40^***^ 0.50^***^

SRS 0.68^***^ 0.67^***^ 0.67^***^ 0.89^***^

Inattention 0.79^***^ 0.53^***^ 0.68^***^

Hyper-Imp 0.44^***^ 0.72^***^

Anxiety --- 0.73^***^

** p<.01, *** p<.001

WASI = Wechsler Abbreviated Scale of Intelligence; FSIQ = Full Scale IQ; ABC = Vineland Adaptive Behavior Composite, SRS = Social Responsiveness Scale Total score, Inattention = Conners Inattention Total score, Hyper-Imp = Conners Hyperactivity/Impulsivity Total score, Anxiety = Spence Children’s Anxiety Scale-Total Total score

**Appendix S3**

**Statistical Analysis of Mid-Childhood LPA Classes on Indicator Variables**

As shown in the main paper (Table 3), ANOVA and Tukey-Kramer post-hocs (*p* < .05) for class differences are, as expected, in line with class identification. For WASI FSIQ the between group ANOVA was significant (﻿*F*(6, 218) = 8.87, *p* < .001) and Tukey-Kramer post-hocs indicated that The Autism + Low IQ class had lower FSIQ than the other classes. For the Vineland ABC (﻿*F*(6, 215) = 38.17, *p* < .001) and the Typically Developing + High IQ class had higher scores than all other classes except the High Anxiety class; the Autism + Low IQ class had lower scores than all other classes except the Autism + Low Adaptive Behaviour class; the Autism + Low Adaptive Behaviour class had lower scores than all other classes except the Autism + Low IQ class. For the SRS (﻿*F*(6, 237) = 312.71, *p* < .001); Conners Inattention (﻿*F*(6, 240) = 86.18, *p* < .001); Conners Hyperactivity/Impulsivity (﻿*F*(6, 240) = 107.81, *p* < .001) and Spence Children’s Anxiety Scale (﻿*F*(6, 216) = 62.14, *p* < .001) and many of the class vs. class differences were significant (see Table 3 for summary of post-hocs).

**Table S7** *Three Year Scores by Mid-Childhood LPA Classes*

|  | TF + High IQ | TF | | | High Anxiety | | | High ADHD | | | Moderate Autism/ADHD | | | | Autism + LAF | | | Autism + Low IQ | | |
| --- | --- | --- | --- | --- | --- | --- | --- | --- | --- | --- | --- | --- | --- | --- | --- | --- | --- | --- | --- | --- |
|  | *Mean (SD)* | *Mean (SD)* | | | *Mean (SD)* | | | *Mean (SD)* | | | *Mean (SD)* | | | | *Mean (SD)* | | | *Mean SD)* | | |
| Age (months) | 37.81 (1.56)  N=70 | 38.16 (3.16)  N=56 | | | 38.00 (1.63)  N=22 | | | 38.26 (2.22)  N=23 | | | 37.58 (1.61)  N=24 | | | | 37.39 (2.12)  N=31 | | | 37.96 (1.90)  N=24 | | |
| Mullen ELC | 119.3 (17.03)_a_  N=70 | 111.55 (18.41)_a_  N=56 | | | 108.50 (20.22)  N=22 | | | 110.74 (18.68)  N=23 | | | 105.67 (23.88)  N=24 | | | | 101.97 (23.72)_c_  N=30 | | | 94.88 (24.50)_b_  N=24 | | |
| Vineland ABC | 104.96 (8.56)_a_  N=68 | 98.75 (10.20)_b,c_  N=55 | | | 97.14 (12.41)_c_  N=21 | | | 99.83 (9.13)_c_  N=23 | | | 96.33 (12.38)_b,c_  N=21 | | | | 93.61 (15.62)_b_  N=31 | | | 85.68 (15.03)_b_  N=25 | | |
| ADOS | 2.66 (2.12)  N=70 | 2.75 (2.35)  N=57 | | | 2.59 (2.11)  N=22 | | | 1.96 (1.87)  N=23 | | | 2.75 (2.54)  N=24 | | | | 2.97 (2.16)  N=32 | | | 2.52 (2.28)  N=25 | | |
| SCQ | 2.38 (2.13)_a_  N=69 | 3.20 (2.63)_a_  N= 56 | | | 4.30 (4.87)_a_  N=20 | | | 4.36 (5.53)_a_  N=22 | | | 5.21 (5.55)_a_  N=24 | | | | 9.94 (8.11)_c_  N=28 | | | 15.90 (8.34)_b_  N=21 | | |
| CBCL Anxiety | 50.47 (1.50)_a,d_  N=43 | 51.19 (2.64)_a_  N=31 | | | 54.38 (6.51)_a_  N=16 | | | 53.23 (5.69)_a_  N=13 | | | 53.28 (6.20)_a_  N=18 | | | | 56.48 (8.28)_a_,_c_  N=23 | | | 63.00 (13.68)_b_  N=18 | | |
| CBCL ADHD | 50.95 (2.68)_a_  N=43 | 50.97 (2.20)_a_  N=31 | | | 52.50 (4.23)_a_  N=16 | | | 57.85 (7.44)_b_  N=13 | | | 53.89 (5.69)_a_  N=18 | | | | 59.91 (7.81)_b_  N=23 | | | 63.61 (9.13)_b_  N=18 | | |
| CBQ Surgency | 4.60 (0.65)  N=68 | 4.53 (0.75)  N=56 | | | 4.45 (0.71)  N=20 | | | 4.86 (0.75)  N=22 | | | 4.72 (0.87)  N=24 | | | | 4.68 (0.62)  N=27 | | | 4.30 (1.06)  N=20 | | |
| CBQ Negative Affect | 3.62 (0.74)_a_  N=68 | 3.71 (0.70)_a,c_  N=56 | | | 4.16 (0.67)  N=20 | | | 4.12 (0.67)  N=22 | | | 3.92 (0.83)d  N=24 | | | | 4.40 (0.82)_b_  N=27 | | | 4.69 (0.92)_b_  N=20 | | |
| CBQ Effortful Control | 5.16 (0.67)_a_  N=68 | 4.98 (0.63)  N=56 | | | 4.97 (0.44)  N=20 | | | 4.90 (0.58)  N=22 | | | 4.81 (0.76)  N=24 | | | | 4.61 (0.93)_b_  N=27 | | | 4.86 (1.07)  N=20 | | |
|  |  | |  |  | |  |  | |  |  | |  |  |  | |  |  | |  |  |

**Note:** Groups marked with different subscript letters (a, b, c) differed significantly with Tukey-Kramer HSD correction applied (p < .05).

TF + High IQ = Typically Functioning + High IQ; TF = Typically Functioning; High Anxiety = High Anxiety traits; High ADHD = High ADHD traits; Moderate Autism/ADHD = Elevated Autism + ADHD traits; Autism + LAF = Autism + Low Adaptive Behaviour; Autism + Low IQ = Autism + Low IQ

**Appendix S4**

**Developmental and behavioural characterisation of the mid-childhood LPA classes at 3-years**

As shown in Table S6, the Autism + Low IQ and Autism + LAF classes had low developmental 3-year scores on the Mullen and Vineland and high levels of autism, ADHD and anxiety traits. There were few differences in the children in the two classes who went on to have elevated mid-childhood ADHD and anxiety traits in isolation, although those with elevated ADHD mid-childhood traits had slightly elevated ADHD scores at 3-years.

**Appendix S5**

**Statistical Analysis of 3-Year Scores by Mid-Childhood LPA Classes**

As shown in Table S5, ANOVA and Tukey-Kramer post-hocs (*p* < .05) indicate that for Mullen ELC (﻿*F*(6, 242) = 5.81, *p* < .001) with the Typically Functioning + High IQ and Typically Functioning classes having higher scores than the Autism + Low IQ class and the Typically Functioning + High IQ class higher scores than the Autism + Low Adaptive Behaviour class. For the Vineland ABC (﻿*F*(6, 237) = 9.92, *p* < .001) the Typically Functioning + High IQ class had higher scores than all other classes except the High ADHD and Moderate Autism/ADHD classes and the Autism + Low IQ class lower scores than all classes except the Autism + Low Adaptive Behaviour class. For the ADOS-2 (﻿*F*(6, 246) = 0.53, *p* = .784). For the SCQ (﻿*F*(6, 233) =26.36, *p* < .001) and the Autism + Low IQ and Autism and LAF classes had higher scores than all other groups (see Table S5 for post-hocs). For the CBCL Anxiety scale (﻿*F*(6, 155) =9.26, *p* < .001) and the Autism + Low IQ class had higher scores than all other classes; the Autism + LAF class had higher scores than the TD + High IQ class. For the CBCL ADHD scale (﻿*F*(6, 155) =18.23, *p* < .001) the Autism + Low IQ and Autism and LAF classes had higher scores than most other groups. For CBQ Surgency (﻿*F*(6, 230) =1.36, *p* = .232); CBQ Negative Affect (﻿*F*(6, 230) =8.36, *p* < .001); and CBQ Effortful Control (﻿*F*(6, 230) =2.24, *p* < .05); with post-hocs indicating no class differences on Surgency, the Autism + Low IQ and Autism + LAF classes having higher scores than some other classes on Negative Affect and only the Autism + LAF class having lower Effortful Control than the TD + High IQ class.

In summary, in terms of 3-year behavioural profiles, the Autism + Low IQ and Autism + LAF classes had low 3-year scores on the Mullen ELC and on Vineland adaptive behaviour and high levels of autism symptoms and ADHD and anxiety trait measures, in large part reflecting the fact that these classes contained most children who obtained an autism diagnosis at the 3-year assessment. There were few clear differences in the children in the two classes he went on to have elevated mid-childhood ADHD and anxiety traits in isolation, although those with elevated ADHD mid-childhood traits had somewhat elevated ADHD traits scores at the 3-year assessment.

**Appendix S6**

**3-Year LPA Analysis**

**3-Year Developmental and Behavioural Measures**

***Developmental Ability.*** Early Learning Composite (ELC) from the *Mullen Scales of Early Learning* (Mullen, 1995) was used to measure of developmental ability. *Vineland-II Adaptive Behavior Composite (ABC)* (Sparrow et al., 2005) was used to measure adaptive functioning.

***Autism measures.*** The Autism Diagnostic Observation Schedule-2 (ADOS-2) (Lord et al., 2012), Autism Diagnostic Interview-Revised (ADI-R) , the Social Communication Questionnaire (SCQ) (Lord et al., 1994; Rutter et al., 2003) and the *Social Responsiveness Scale-2* (*SRS-2*) (Constantino & Gruber, 2012) were administered.

***ADHD and Anxiety.*** Emerging ADHD and anxiety traits were measured using the parent-report DSM subscales of the Child Behaviour Checklist-Preschool (CBCL-P 1.5-5) (Achenbach & Rescorla, 2001).

***Temperament.*** Parents completed the *Childhood Behavior Questionnaire* (CBQ (Putnam & Rothbart, 2006)) to measure the temperamental dimensions Surgency, Negative Affect, and Effortful Control.

***Statistical Analysis***

We conducted latent profile analysis (LPA) using continuous indicator variables to identify homogeneous classes based on the following 3-year indicator variables: Mullen, Vineland ABC, SRS-2 total raw score, CBCL ADHD and anxiety raw score. Variables were modelled, conditional on latent class, using Poisson distributions for all variables. LPA was performed using the *gsem* command in Stata 18 (StataCorp., 2023) on the whole sample with at least one of the six outcome measures available (*n* = 263; 256 children had > 3 measures). As described in the main paper; the “best fitting” solution was based on the BIC, ICL and entropy fit statistics; the proportion of participants represented in each class; and the extent to which classes captured clinically meaningful subgroups.

***Results***

We chose the 6-class solution (entropy = 0.85, BIC = 7638.05, ICL = 7752.41) as providing the most robust and clinically meaningful distribution of classes, with a minimum class size comprising 6.4% (N = 15) of the sample and average MAP values for each class all > 0.87.

Based on the pattern across the measures and also the presence vs. absence of a 3-year autism diagnosis we labelled the classes as follows. Class 1 (*N* = 71, 28%) = *Typically Developing + Very High IQ* *(TD + Very High IQ)* (2% have autism); Class 2 (*N* = 86, 34%) = *Typical Development + High IQ (TD + High IQ) (1% have autism)*^[[1]](#footnote-1)^; Class 3 (*N* = 29, 11%) = *Typically Developing (TD)* (10% have autism); Class 4 (*N* = 15, 6%) = *Typical Development + Some Behavioural Concerns (TD + SBC)* (13% have autism); Class 5 (*N* = 29; 11%) = *Autism or Low IQ* (32% have autism); and Class 6 (*N* = 26, 10%) = *Autism + Low Adaptive Functioning (Autism + LAF)* (77% have autism).

3-year developmental and behavioural scores are shown in Table S7; the distribution by family history grouping in Table S8; the 3-Year LPA Classes by Sex and 3-Year Autism Diagnosis in Table S9; and the 3-Year LPA Class by Mid-Childhood LPA Class comparison in Table S10 (see also Figure 2 in main paper). We descriptively highlight patterns of continuity vs. discontinuity between typical and atypical profiles of classes below Table S10 but given the complex 6 x 7 crosstabulation across the 3-Year and Mid-Childhood LPA class solutions do not attempt to conduct any formal statistical tests.

| **Table S8** *3-Years Scores by 3-Year LPA Classes* | | | | | | | | | | | | | | | | | | |
| --- | --- | --- | --- | --- | --- | --- | --- | --- | --- | --- | --- | --- | --- | --- | --- | --- | --- | --- |
|  |  | | | | | | | | | | | | | | | | | |
|  | TD + Very High IQ | | | TD + High IQ | | | TD | | | TD + SBC | | | Autism or Low IQ | | | Autism + LAF | | |
|  | Mean | SD | N | Mean | SD | N | Mean | SD | N | Mean | SD | N | Mean | SD | N | Mean | SD | N |
| Age (months) | 37.79 | (1.66) | 68 | 38.13 | (2.86) | 85 | 37.41 | (1.35) | 29 | 37.87 | (1.36) | 15 | 37.86 | (1.84) | 28 | 37.92 | (2.29) | 25 |
| Mullen ELC | 126.56 | (9.77) | 68 | 117.40 | (11.93) | 85 | 93.83 | (9.05) | 29 | 121.07 | (9.15) | 15 | 74.19 | (11.69) | 28 | 91.21 | (21.73) | 24 |
| Vineland ABC | 107.87 | (7.14) | 63 | 102.01 | (8.48) | 84 | 95.55 | (7.60) | 29 | 95.87 | (6.74) | 15 | 84.18 | (8.65) | 28 | 81.60 | (15.67) | 25 |
| ADOS-2 CSS | 2.20 | (1.81) | 69 | 2.28 | (1.83) | 86 | 3.28 | (2.28) | 29 | 2.27 | (2.12) | 15 | 4.04 | (3.08) | 28 | 3.04 | (2.49) | 26 |
| SCQ | 1.80 | (1.41) | 67 | 3.38 | (2.42) | 78 | 2.59 | (1.80) | 29 | 8.60 | (5.17) | 15 | 7.49 | (6.46) | 27 | 19.69 | (5.80) | 24 |
| SRS T-Score | 39.32 | (1.85) | 63 | 45.21 | (1.97) | 80 | 40.36 | (1.75) | 28 | 56.43 | (4.88) | 14 | 50.29 | (4.25) | 24 | 77.17 | (6.60) | 23 |
| CBCL ADHD | 51.44 | (3.51) | 45 | 51.81 | (3.79) | 47 | 51.00 | (3.00) | 21 | 59.00 | (5.38) | 12 | 57.82 | (6.80) | 17 | 67.20 | (6.81) | 20 |
| CBCL Anxiety | 50.04 | (0.21) | 45 | 52.57 | (4.75) | 47 | 50.48 | (1.21) | 21 | 54.75 | (6.21) | 12 | 55.18 | (6.03) | 17 | 66.65 | (11.94) | 20 |

TD + Very High IQ = Typically Developing + Very High IQ, Autism + LAF = Autism + Low Adaptive Functioning, Autism or Low IQ, TD + SBC = Typical Development + Some Behavioural Concerns, TD = Typically Developing, TD + High IQ = Typically Developing + High IQ

Mullen ELC = Mullen Early Learning Composite, Vineland ABC = Vineland Adaptive Behavior Composite, ADOS-2 CSS = Autism Diagnostic Observation Schedule-2 Calibrated Severity Score, SCQ = Social Communication Questionnaire, SRS = Social Responsiveness Scale, CBCL ADHD T-score, CBCL Anxiety T-score

**Table S9** *3-Year LPA class by Family History Group*

__________________________________________________________________________________

No FH FH-Autism FH-ADHD FH-Autism Total

+ ADHD

*N* = 62 *N* = 129 *N* = 14 *N* = 51 *N* = 256

Row % Row % Row % Row %

Column % Column % Column % Column %

__________________________________________________________________________________

TD + Very High IQ 28 25 8 10 71

39.4% 35.2% 11.3% 14.1%

45.2% 19.4% 57.1% 19.6%

TD + High IQ 28 40 3 15 86

32.6% 46.5% 3.5% 17.4%

45.2% 31.0% 21.4% 29.4%

TD 4 17 0 8 29

13.8% 58.6% 0% 27.6%

6.5% 13.2% 0% 15.7%

TD + SBC 1 8 2 4 15

6.7% 53.3% 13.3% 26.7%

1.6% 6.2% 14.3% 7.8%

Autism or Low IQ 1 20 1 7 29

3.5% 69.0% 3.5% 24.1%

1.6% 15.5% 7.1% 13.7%

Autism + LAF 0 19 0 7 26

0% 73.1% 0% 26.9%

0% 14.7% 0% 13.7%

__________________________________________________________________________________

TD + Very High IQ = Typically Developing + Very High IQ, Autism + LAF = Autism + Low Adaptive Functioning, Autism or Low IQ, TD + SBC = Typical Development + Some Behavioural Concerns, TD = Typically Developing, TD + High IQ = Typically Developing + High IQ

**Table S10** *3-Year LPA Classes by Sex and Mid-Childhood Autism Diagnosis (Early vs. Later)*^[[2]](#footnote-2)^

| LPA class | Male  *N* = 128 | Female  *N* = 128 | Earlier Autism  Diagnosis  *N* = 30 | Later Autism Diagnosis  *N* = 34 | Never Autism^[[3]](#footnote-3)^  *N* = 157 |
| --- | --- | --- | --- | --- | --- |
|  | *N (Row %)* | *N (Row %)* | *N (Row %)* | *N (Row %)* | *N (Row %)* |
| TD + Very High IQ | 46 (65%) | 25 (35%) | 1 (2%) | 4 (7%) | 56 (98%) |
| TD + High IQ | 47 (55%) | 39 (45%) | 0 (0%) | 10 (14%) | 64 (86%) |
| TD | 10 (34%) | 19 (66%) | 2 (8%) | 5 (20%) | 18 (72%) |
| TD + SBC | 6 (40%) | 9 (60%) | 2 (15%) | 6 (46%) | 5 (38%) |
| Autism or Low IQ | 8 (28%) | 21 (72%) | 7 (28%) | 7 (28%) | 11 (42%) |
| Autism + LAF | 11 (42%) | 15 (58%) | 18 (78%) | 2 (9%) | 3 (13%) |

TD + Very High IQ = Typically Developing + Very High IQ; TD + High IQ = Typically Developing + High IQ; TD = Typically Developing; TD + SBC = Typical Development + Some Behavioural Concerns; Autism or Low IQ; Autism + LAF = Autism + Low Adaptive Functioning

**Table S11** *3-Year LPA Class by Mid-Childhood LPA Class*

_____________________________________________________________________________________________________________________

*Mid-Childhood* TF TF High Anxiety High ADHD Moderate Autism + LAF Autism + Low IQ

*LPA class* + High IQ Autism/ADHD

N N N N N N N

Row % Row % Row % Row % Row % Row % Row %

Column % Column % Column % Column % Column % Column % Column %

______________________________________________________________________________________________________________________

*3-Year LPA class*

TD + Very High IQ 36 18 6 3 6 2 0

50.7% 25.4% 8.5% 4.2% 8.5% 2.8% 0%

51.4% 30.5% 27.3% 12.5% 25.0% 6.3% 0%

TD + High IQ 20 26 9 13 6 8 4

23.3% 30.2% 10.5% 15.1% 7.0% 9.3% 4.7%

28.6% 44.1% 40.9% 54.2% 25.0% 25.0% 16.0%

TD 12 7 2 3 1 3 1

41.4% 24.1% 6.9% 10.3% 3.5% 10.3% 3.5%

17.1% 11.9% 9.1% 12.5% 4.2% 9.4% 4.0%

TD + SBC 1 2 2 1 3 5 1

6.7% 13.3% 13.3% 6.7% 20.0% 33.3% 6.7%

1.4% 3.4% 9.1% 4.2% 12.5% 15.6% 4.0%

Autism or Low IQ 1 6 2 4 7 5 4

3.5% 20.7% 6.9% 13.8% 24.1% 17.2% 13.8%

1.4% 10.2% 9.1% 16.7% 29.2% 15.6% 16.0%

Autism + LAF 0 0 1 0 1 9 15

0% 0% 3.9% 0% 3.9% 34.6% 57.7%

0% 0% 4.6% 0% 4.2% 28.0% 60.0%

___________________________________________________________________________________________________________________________

Mid-Childhood LPA classes: TF + High IQ = Typically Functioning + High IQ; TF = Typically Functioning; High Anxiety = High Anxiety traits; High ADHD = High ADHD traits; Moderate Autism/ADHD = Elevated Autism + ADHD traits; Autism + LAF = Autism + Low Adaptive Behaviour; Autism + Low IQ = Autism + Low IQ

3-Year LPA classes: TD + Very High IQ = Typically Developing + Very High IQ; TD + High IQ = Typically Developing + High IQ; TD = Typically Developing; TD + SBC = Typical Development + Some Behavioural Concerns; Autism or Low IQ; Autism + LAF = Autism + Low Adaptive Functioning

**Appendix S7**

**Mid-Childhood LPA repeated for autism and /or ADHD family history infants only**

We repeated the LPA with the autism and /or ADHD family history infants only (*N* = 132 FH-Autism, *N* = 15 FH-ADHD, *N* = 51 FH-Autism + ADHD) with the same mid-childhood indicator variables (identify homogeneous classes based on the following mid-childhood indicator variables: WASI FSIQ, Vineland ABC, SRS-2 total raw score, Conners-3 Inattention and Hyperactivity/Impulsivity raw scores and SCAS total anxiety raw score).

The 6-class solution provided the most robust and clinically meaningful distribution of classes (entropy = 0.89, BIC = 8646.97, ICL = 8681.66), with a minimum class size comprising 9.1% (N = 17) of the sample and average MAP values for all classes > 0.93. We labelled the classes as follows:

Class 1 (N = 39, 20%) = Typically Functioning + High IQ (TF + High IQ); Class 2 (N = 29, 15%) = Autism + Low IQ (Autism + Low IQ); Class 3 (N = 30; 15%) = High ADHD; Class 4 (N = 28, 14%) = Autism + Low Adaptive Function (Autism + LAF); Class 5 (N = 55, 28%) = Typically Functioning (TF); and Class 6 (N = 17, 9%) = Elevated Anxiety.

The LPA including autism and / or ADHD family history infants only produced a similar 6-class solution to the main LPA that included No-FH infants. There were two typically developing /functioning classes, two predominantly autism classes (one with low IQ and low adaptive functioning, one low adaptive functioning), and two classes with, respectively, predominantly isolated ADHD and anxiety traits in isolation. Children with an autism diagnosis were found in all classes. Sex was balanced across all classes.

Scores on the indicator variables for classes are shown in Table S11 and the distribution of classes by family history group in Table S12 and by autism diagnosis and sex in Table S13. The most notable difference from the main LPA including the No-FH infants is that the High ADHD and Elevated Anxiety classes had a higher proportion of children with an autism diagnosis (but still a minority – See Table S13).

| **Table S12** *Mid-Childhood Scores by LPA Classes for Family History Infants only* | | | | | | | | | | | | | | | | | | |
| --- | --- | --- | --- | --- | --- | --- | --- | --- | --- | --- | --- | --- | --- | --- | --- | --- | --- | --- |
|  |  | | | | | | | | | | | | | | | | | |
|  | TF + High IQ | | | TF | | | Elevated Anxiety | | | High ADHD | | | Autism + LAF | | | Autism + Low IQ | | |
|  | Mean | SD | N | Mean | SD | N | Mean | SD | N | Mean | SD | N | Mean | SD | N | Mean | SD | N |
| Age (months) | 102.7 | (13.4) | 39 | 106.38 | (16.12) | 53 | 115.82 | (14.97) | 17 | 106.6 | (14.3) | 29 | 101.07 | (14.74) | 28 | 106.7 | (17.5) | 28 |
| WASI FSIQ | 113.24 | (14.13) | 33 | 111.49 | (15.30) | 49 | 110.06 | (10.43) | 16 | 106.65 | (17.97) | 23 | 110.57 | (12.28) | 21 | 90.09 | (14.67) | 23 |
| Vineland ABC | 109.26 | (11.07) | 34 | 102.00 | (9.01) | 48 | 98.69 | (13.07) | 16 | 94.09 | (11.92) | 23 | 82.32 | (13.87) | 22 | 77.70 | (12.72) | 20 |
| SRS | 41.24 | (1.86) | 34 | 48.02 | (2.77) | 50 | 59.75 | (6.64) | 16 | 53.60 | (4.74) | 30 | 75.31 | (8.25) | 26 | 84.92 | (12.28) | 25 |
| Inattention | 42.91 | (3.85) | 35 | 49.75 | (7.21) | 52 | 51.12 | (8.64) | 16 | 69.82 | (11.19) | 28 | 70.37 | (11.92) | 27 | 77.71 | (12.39) | 24 |
| Hyper-Imp | 44.91 | (5.33) | 35 | 49.12 | (6.39) | 52 | 52.12 | (6.24) | 16 | 71.57 | (12.36) | 28 | 76.48 | (10.56) | 27 | 77.04 | (12.24) | 24 |
| Anxiety | 48.09 | (6.58) | 34 | 51.92 | (6.50) | 48 | 62.07 | (7.04) | 14 | 50.09 | (8.30) | 22 | 55.73 | (5.85) | 22 | 68.82 | (2.97) | 22 |

TF + High IQ = Typically Functioning + High IQ; Autism + Low IQ = Autism + Low IQ; High ADHD = High ADHD traits; Autism + LAF = Autism + Low Adaptive Behaviour; TF = Typically Functioning; Elevated Anxiety = Elevated Anxiety traits

WASI = Wechsler Abbreviated Scale of Intelligence; FSIQ = Full Scale IQ; ABC = Vineland Adaptive Behavior Composite, SRS = Social Responsiveness Scale T-score, Inattention = Conners Inattention T-score, Hyper-Imp = Conners Hyperactivity/Impulsivity T-score, Anxiety = Spence Children’s Anxiety Scale-Total T-score

**Table S13** *Mid-Childhood classes for Family History Infants only LPA by Family History Group*

FH-Autism FH-ADHD FH-Autism + ADHD

*N* = 132 *N* = 15 *N* = 51

Row % Row % Row %

Column % Column % Column %

TF + High IQ 28 3 8

72% 8% 21%

21% 20% 16%

TF 43 2 10

78% 4% 18%

33% 13% 20%

Elevated Anxiety 12 1 4

71% 6% 24%

9% 7% 8%

High ADHD 15 7 8

50% 23% 27%

11% 47% 16%

Autism + LAF 13 1 14

47% 4% 50%

10% 7% 27%

Autism + Low IQ 21 1 7

72% 4% 24%

16% 7% 14%

TF + High IQ = Typically Functioning + High IQ; Autism + Low IQ = Autism + Low IQ; High ADHD = High ADHD traits; Autism + LAF = Autism + Low Adaptive Behaviour; TF = Typically Functioning; Elevated Anxiety = Elevated Anxiety traits

**Table S14** *Mid-Childhood classes for Family History Infants only LPA by Autism Diagnosis and by Sex*

|  | LPA class | Sex  Male  *N* = 99 | Female  *N* = 99 | Earlier Autism Diagnosis^a^  *N* = 30 | Later Autism Diagnosis  *N* = 32 | Never Autism  *N* = 101 |
| --- | --- | --- | --- | --- | --- | --- |
|  |  | *N (Row %)* | *N (Row %)* | *N (Row %)* | *N (Row %)* | *N (Row %)* |
|  | TF + High IQ | 19 (49%) | 20 (51%) | 1 (3%) | 1 (3%) | 58 (94%) |
|  | TF | 28 (51%) | 27 (49%) | 3 (6%) | 6 (13%) | 37 (80%) |
|  | Elevated Anxiety | 8 (47%) | 9 (53%) | 2 (13%) | 3 (20%) | 10 (67%) |
|  | High ADHD | 12 (40%) | 18 (60%) | 3 (13%) | 3 (13%) | 18 (75%) |
|  | Autism + LAF | 17 (61%) | 11 (39%) | 10 (45%) | 9 (41%) | 3 (14%) |
| Autism + Low IQ | | 15 (52%) | 14 (48%) | 11 (46%) | 10 (42%) | 3 (13%) |

a = Diagnostic assessment only completed on *N* = 163 children (excluding *N* = 3 children who ‘lost diagnosis’)

TF + High IQ = Typically Functioning + High IQ; Autism + Low IQ = Autism + Low IQ; High ADHD = High ADHD traits; Autism + LAF = Autism + Low Adaptive Behaviour; TF = Typically Functioning; Elevated Anxiety = Elevated Anxiety traits

**References**

Achenbach, T. M., & Rescorla, L. A. (2001). *Manual for the ASEBA School-Age Forms & Profiles.* University of Vermont, Research Center for Children, Youth, & Families.

Conners. (2009). *Conners Early Childhood Manual.* Multi-Health Systems Inc.

Conners, C. (2008). *Conners 3rd Edition manual.* Toronto, Ontario, Canada: Multi-Health Systems.

Conners, C., Erdhardt, D., & Sparrow, E. (1999). *Conners Adults ADHD Ratings Scales (CAARS).* Multi-Health Systems Inc.

Constantino, J., & Gruber, C. (2012). *Social Responsiveness Scale, 2nd Edn (SRS-2).* Western Psychological Services, Torrance, CA.

Goodman, R., Ford, T., Richards, H., Gatward, R., & Meltzer, H. (2000). The Development and Well-Being Assessment: Description and initial validation of an integrated assessment of child and adolescent psychopathology. *Journal of Child Psychology and Psychiatry, and Allied Disciplines*, *41*(5), 645–655.

Lord, C., Rutter, M., DiLavore, P., Risi, S., Gotham, K., & Bishop, S. (2012). *Autism Diagnostic Observation Schedule, second edition (ADOS-2).* Western Psychological Services.

Lord, C., Rutter, M., & Le Couteur, A. (1994). Autism Diagnostic Interview-Revised: A revised version of a diagnostic interview for caregivers of individuals with possible pervasive developmental disorders. *Journal of Autism and Developmental Disorders*, *24*(5), 659–685. https://doi.org/10.1007/BF02172145

Mullen, E. (1995). *Mullen Scales of Early Learning.* Minnesota: American Guidance Service.

Putnam, S. P., & Rothbart, M. K. (2006). Development of short and very short forms of the Children’s Behavior Questionnaire. *Journal of Personality Assessment*, *87*(1), 102–112. https://doi.org/10.1207/s15327752jpa8701_09

Russell, G., Rodgers, L. R., Ukoumunne, O. C., & Ford, T. (2014). Prevalence of parent-reported ASD and ADHD in the UK: Findings from the Millennium Cohort Study. *Journal of Autism and Developmental Disorders*, *44*(1), 31–40. https://doi.org/10.1007/s10803-013-1849-0

Rutter, M., Bailey, A., & Lord, C. (2003). *The Social Communication Questionnaire (SCQ).* Los Angeles, CA: Western Psychological Services.

Simonoff, E., Pickles, A., Charman, T., Chandler, S., Loucas, T., & Baird, G. (2008). Psychiatric disorders in children with autism spectrum disorders: Prevalence, comorbidity, and associated factors in a population-derived sample. *Journal of the American Academy of Child and Adolescent Psychiatry*, *47*(8), 921–929. https://doi.org/10.1097/CHI.0b013e318179964f

Sparrow, S., Cicchetti, D., & Balla, D. (2005). *Vineland Adaptive Behavior Scales, Second edition (Vineland-II).* Circle Pines, MN: America Guidance Service.

StataCorp. (2023). *Stata Statistical Software: Release 18.* StataCorp LLC.

1. We label the classes here differently from a previous 3-Year LPA analysis that used overlapping but different indicator variables on part but not all of the current sample reported in Charman et al (2023) to align the class labels more closely to those with have adopted in the current mid-childhood LPA analysis. [↑](#footnote-ref-1)
2. Only includes children who completed a full autism diagnostic assessment at Mid-Childhood (N = 222) [↑](#footnote-ref-2)
3. Excludes the N=3 children who ‘lost diagnosis’ between 3-Years and Mid-Childhood (see Bazelmans et al., 2024) [↑](#footnote-ref-3)
